# New Bipolar Host Materials Based on Indolocarbazole for Red Phosphorescent OLEDs

**DOI:** 10.3390/ma17174347

**Published:** 2024-09-02

**Authors:** Sunwoo Park, Hyukmin Kwon, Sangwook Park, Saeyoung Oh, Kiho Lee, Hayoon Lee, Seokwoo Kang, Dongmin Park, Jongwook Park

**Affiliations:** 1 Integrated Engineering, Department of Chemical Engineering, Kyung Hee University, Yongin 17104, Republic of Korea; mdrafix@khu.ac.kr (S.P.); hm531@khu.ac.kr (H.K.); pswook@khu.ac.kr (S.P.); tpdud5821@khu.ac.kr (S.O.); kiholee@khu.ac.kr (K.L.); kssarang1@khu.ac.kr (H.L.); swkang@khu.ac.kr (S.K.); 2 Center for Supramolecular Optoelectronic Materials (CSOM), Department of Materials Science and Engineering, Seoul National University, 1 Gwanak-ro, Gwanak-gu, Seoul 08826, Republic of Korea; parkdm0411@gmail.com

**Keywords:** OLED emitter, bipolar host, indolocarbazole, diphenyltriazine, red phosphorescence

## Abstract

We designed and synthesized new indolocarbazole-triazine derivatives, 9-di-tert-butyl-5,7-bis(4-(4,6-diphenyl-1,3,5-triazin-2-yl)phenyl)-5,7-dihydroindolo[2,3-b]carbazole (2TRZ-P-ICz) and 3,9-di-tert-butyl-5,7-bis(5′-(4,6-diphenyl-1,3,5-triazin-2-yl)-[1,1′:3′,1″-terphenyl]-2′-yl)-5,7-dihydroindolo[2,3-b]carbazole (2TRZ-TP-ICz), as new bipolar host materials for red phosphorescent OLEDs. In the film state, 2TRZ-P-ICz and 2TRZ-TP-ICz exhibited photoluminescence maxima at 480 nm and 488 nm, respectively. The dipole moment characteristics of the new compounds under various solvent conditions were investigated using the Lippert–Mataga equation. The results showed that the dipole moment of 2TRZ-P-ICz is 26.9D, while that of 2TRZ-TP-ICz is 21.3D. The delayed fluorescence lifetimes were 0.188 μs for 2TRZ-P-ICz and 2.080 μs for 2TRZ-TP-ICz, with 2TRZ-TP-ICz showing TADF characteristics. Additionally, 2TRZ-TP-ICz was found to have a ΔEST of less than 0.2 eV. The triplet energy levels of the newly synthesized bipolar host materials were found to be 2.72 and 2.75 eV, confirming their suitability for use in red phosphorescent OLEDs. To investigate the carrier mobility of the synthesized materials, hole-only devices and electron-only devices were fabricated and tested. The hole mobility value at 1V was found to be 3.43 × 10^−3^ cm^2^/Vs for 2TRZ-P-ICz and 2.16 × 10^−3^ cm^2^/Vs for 2TRZ-TP-ICz. For electron mobility at 1V, 2TRZ-P-ICz showed a value of 4.41 × 10^−9^ cm^2^/Vs, while 2TRZ-TP-ICz exhibited a value of 9.13 × 10^−9^ cm^2^/Vs. As a result, when the new material was used as a host in red phosphorescent OLEDs, 2TRZ-TP-ICz achieved a current efficiency of 9.92 cd/A, an external quantum efficiency of 13.7%, CIE coordinates of (0.679, 0.319), and an electroluminescence maximum wavelength of 626 nm.

## 1. Introduction

Organic light-emitting diodes (OLEDs) generally use emitters classified into fluorescence and phosphorescence based on their emission mechanisms [1,2,3]. Fluorescent materials utilize only singlet excitons, leading to an internal quantum efficiency (IQE) of only 25% [4]. In contrast, phosphorescent materials can theoretically utilize both singlet and triplet excitons, achieving an internal quantum efficiency of up to 100% and thereby enabling higher luminous efficiency [5]. To realize high efficiency, a key characteristic of phosphorescent host materials is that they must possess an appropriate triplet level to facilitate effective energy transfer to the phosphorescent dopant. Secondly, it should have appropriate highest occupied molecular orbital (HOMO) and lowest unoccupied molecular orbital (LUMO) levels to lower the energy barrier between adjacent layers, enabling efficient charge injection. Thirdly, they should exhibit suitable bipolar characteristics to facilitate effective carrier recombination and transport. Lastly, the materials need to have a high thermal stability to prevent degradation during device operation, thereby contributing to enhanced efficiency and longevity of the device. To address these issues, we selected indolocarbazole (ICz) as the donor group and diphenyltriazine (TRZ) as the acceptor group, incorporating either a phenyl or a larger terphenyl (TP) linker between the two moieties to synthesize two new host materials with bipolar characteristics. ICz, compared to carbazole, has a relatively strong electron-donating effect and offers a high photoluminescence quantum yield (PLQY), excellent thermal stability, and structural diversity due to its superior carrier mobility and rigidity [6,7,8]. TRZ, on the other hand, possesses excellent thermal and chemical stability, appropriate electron affinity, and features of diphenyl groups that can exhibit steric effects, making it easy to adjust the energy levels of TADF structures and preventing quenching caused by intermolecular aggregation [9,10,11]. Using bulky linkers such as TP and highly substituted phenyl moieties can suppress intramolecular rotation and vibration, thereby achieving a high triplet energy level. This molecular design strategy is applied to various TADF dopants. The Chihaya Adachi group reported a multiple donor–acceptor type of TADF emitter that incorporates multiple donors and acceptors into a central phenyl ring [12]. These emitters enhance TADF properties and improve the stability of the excited state by suppressing intramolecular rotation and vibration through the incorporation of electron-donating carbazole and 9,10-dihydro-9,9-dimethylacridine units, thereby maximizing charge transfer (CT) characteristics. The Jun Yeob Lee group reported a dual-core type TADF emitter, which incorporates multiple TADF emitters with a single core. This approach enhances optical absorption through the introduction of various chromophores and improves PLQY [13]. Additionally, the Chihaya Adachi group reported on a type of TADF emitter that incorporates multiple donors into a central benzonitrile (BN) unit [14]. Their results show that asymmetrically introducing various donors significantly twists the molecular dihedral angle, thereby suppressing aggregation. The incorporation of bulky donors prevents intermolecular aggregation quenching, which enhances TADF properties and results in a ΔE_ST_ of less than 0.2 eV and a large k_RISC_. Furthermore, this molecular design can induce TADF, and using materials with TADF characteristics as hosts can reduce non-radiative decay pathways from the triplet state of the host. This maximizes energy transfer and enhances device efficiency. Such device architectures have been under continuous investigation since the development of TADF. The Lian Duan group reported on a TADF host based on indenocarbazole–triazine, named DMIC-TRZ, which takes into account both charge mobility and charge injection [15]. The study revealed that DMIC-TRZ possesses bipolar characteristics, providing balanced charge transport capabilities and efficient Förster and Dexter energy transfer. This leads to a reduction in singlet–triplet and triplet–triplet annihilation processes. As a result, the orange phosphorescent OLED device utilizing DMIC-TRZ exhibited an external quantum efficiency (EQE) of 23.2%. The Junyeob Lee group reported on BO-Cz-Si-1 and BO-Cz-Si-2, which are TADF hosts based on 5,9-dioxa-13b-boranaphtho[3,2,1-de] anthracene (DOBNA) [16]. The DOBNA derivatives exhibited excellent TADF characteristics and polaron stability due to their high triplet energy, effectively suppressing slow triplet exciton-related quenching processes. Consequently, the blue phosphorescent OLED using BO-Cz-Si-1 demonstrated an EQE of 23.5% and CIE coordinates of (0.13, 0.18).

In this study, we investigated the photophysical, thermal, and electroluminescence properties of 3,9-di-tert-butyl-5,7-bis(4-(4,6-diphenyl-1,3,5-triazin-2-yl)phenyl)-5,7-dihydroindolo[2,3-b]carbazole (2TRZ-P-Cz) and 3,9-di-tert-butyl-5,7-bis(5′-(4,6-diphenyl-1,3,5-triazin-2-yl)-[1,1′:3′,1″-terphenyl]-2′-yl)-5,7-dihydroindolo[2,3-b]carbazole (2TRZ-TP-Cz), which were synthesized using the aforementioned molecular design strategy. Additionally, we compared and analyzed the changes in OLED characteristics based on the type of linker.

## 2. Materials and Methods

### 2.1. Materials and Instrumentation

All reagents and solvents were purchased as reagent grade and used without further purification. Analytical thin-layer chromatography (TLC) was conducted using Merck 60 F254 silica gel plates, and column chromatography was performed using Merck 60 silica gel (Burlington, MA, USA), (230–400 mesh). ^1^H and ^13^C NMR spectra were recorded in chloroform-d (CDCl_3_) and dimethylsulfoxide (DMSO-d_6_) using a JNM-ECZ400S/L1 NMR spectrometer (JEOL, Tokyo, Japan) at ambient temperature. High-resolution mass spectrometry (HRMS) was performed using fast atom bombardment (FAB) using a JMS-700, 6890 Series mass spectrometer (JEOL, Tokyo, Japan). Optical UV–vis absorption spectra were recorded using a UV-1900i UV/Vis/NIR spectrometer (Shimadzu, Kyoto, Japan). Photoluminescence (PL) spectroscopy was conducted using a PerkinElmer LS55 luminescence spectrometer with an Xe flash lamp (PerkinElmer, Inc., Waltham, MA, USA). Absolute photoluminescence quantum yield (PLQY) measurements were carried out using a Hamamatsu Quantaurus-QY C11347 Absolute PL Quantum Yield spectrometer (Hamamatsu Photonics, Shizuoka-ken, Japan). Transient photoluminescence was measured using a Quantaurus-Tau fluorescence lifetime measurement system (Hamamatsu Photonics, Shizuoka-ken, Japan). Density functional theory (DFT) calculations for 2TRZ-P-ICz and 2TRZ-TP-ICz were performed using the B3LYP-D3 functional and the def2-TZVPP basis set using the ORCA program package (V 5.0.4) [17]. The glass transition temperature (T_g_), melting temperature (T_m_), and crystallization temperature (T_c_) of the compounds were determined using differential scanning calorimetry (DSC) under a nitrogen atmosphere using a DSC 26 (TA Instruments, New Castle, DE, USA). Degradation temperatures (T_d_) were measured based on a thermogravimetric analysis (TGA) using a SDT Q600 (TA Instruments, New Castle, DE, USA), with samples heated to 700 °C at a rate of 10 °C/min. The HOMO energy levels were determined using ultraviolet photoelectron yield spectroscopy (Riken Keiki AC-2, Nara, Japan), and the LUMO energy levels were derived from the HOMO energy levels and the bandgaps. Film samples of the synthesized compounds were deposited to a thickness of 50 nm at a deposition rate of 1 Å/s under a vacuum of 10^−6^ torr. For electroluminescence (EL) devices, all organic layers were deposited under the same vacuum conditions at a rate of 1 Å/s to cover an area of 4 mm^2^. LiF and Al layers were deposited continuously under these vacuum conditions. The current–voltage–luminance (I–V–L) properties of the fabricated EL devices were measured using a Keithley 2400 electrometer (Tektronix, Cleveland, OH, USA), and the light intensities were measured using a Minolta CS-1000A (Konica Minolta, Tokyo, Japan). The devices were stored in a glovebox to maintain stability against moisture and air.

### 2.2. Synthesis

#### 2.2.1. Synthesis of 1,5-Dibromo-2,4-dinitrobenzene, (**1**)

In a 100 mL round-bottom flask, a mixture of sulfuric acid (10 mL) and nitric acid (10 mL) was prepared and stirred for 10 min. To this mixture, 1,3-dibromobenzene (10.7 g, 45.3 mmol) was added dropwise over 15 min while maintaining the temperature at 0 °C using an ice bath. Following the completion of the addition, the reaction mixture was allowed to warm to room temperature for 30 min. The ice bath was then replaced with an oil bath, and the reaction temperature was increased to 50 °C. The mixture was stirred at this temperature for 2 h. Upon completion of the reaction, the mixture was poured into ice-cold water (0 °C) and stirred slowly for 30 min. The resulting solid precipitate was filtered, washed with deionized water, and then recrystallized from a mixture of ethanol and acetone. The recrystallized product was washed with methanol, yielding 1,5-dibromo-2,4-dinitrobenzene (12.0 g, 36.5 mmol) with a yield of 82%. ^1^H NMR (400 MHz, Chloroform-d, δ): 8.45 (s, 1H), 8.22 (s, 1H).

#### 2.2.2. Synthesis of 1,5-Dibromo-2,4-dinitrobenzene, (**2**)

In a 500 mL round-bottom flask, 1-bromo-4-(tert-butyl)benzene (10.0 mL, 57.5 mmol) and bis(pinacolato)diboron (17.5 g, 69.0 mmol) were dissolved in 200 mL of toluene. To this solution, potassium acetate (8.45 g, 86.1 mmol) and [1,1′-bis(diphenylphosphino)ferrocene]palladium(II) dichloride (Pd(dppf)Cl_2_) (1.12 g, 2.31 mmol) were added. The reaction mixture was then stirred under a nitrogen atmosphere at 90 °C for 16 h. After the reaction was complete, the mixture was allowed to cool to room temperature and was subsequently extracted using ethyl acetate (EA) and deionized water. The organic layer was dried over anhydrous magnesium sulfate (MgSO_4_) and filtered. The resulting product was purified using silica gel column chromatography using n-hexane as the eluent, yielding a white solid (13.2 g, 50.7 mmol) with a yield of 88%. ^1^H NMR (400 MHz, chloroform-*d*, δ): 7.86–7.76 (m, 2H), 7.48–7.41 (m, 2H), 1.36 (d, *J* = 3.2 Hz, 21H).

#### 2.2.3. Synthesis of 1,5-Dibromo-2,4-dinitrobenzene, (**3**)

In a 100 mL round-bottom flask, compound (**1**) (3 g, 0.9 mmol), compound (**2**) (0.62 g, 1.98 mmol), 6 mL of 2 M potassium carbonate (K_2_CO_3_) aqueous solution, and 0.051 g (0.044 mmol) of tetrakis(triphenylphosphine)palladium(0) (Pd(PPh_3_)_4_) were added and dissolved in 50 mL of toluene. The reaction mixture was stirred at 90 °C under a nitrogen atmosphere for 16 h. Upon completion, the mixture was cooled to room temperature and quenched with deionized water. The resulting mixture was extracted using EA and deionized water. The organic layer was dried over anhydrous MgSO_4_ and filtered. The residue was purified using silica gel column chromatography using a 1:10 mixture of ethyl acetate and n-hexane as the eluent, yielding a yellow solid (3.22 g, 7.45 mmol) with a yield of 81%. ^1^H NMR (400 MHz, Chloroform-*d*, δ): 8.38 (s, 1H), 7.55 (s, 1H), 7.47–7.44 (m, 4H), 7.29–7.26 (m, 4H), 1.34 (s, 18H).

#### 2.2.4. Synthesis of 1,5-Dibromo-2,4-dinitrobenzene, (**4**)

In a 100 mL round-bottom flask, compound (**3**) (10.0 g, 1.36 mmol), triethylphosphite (23 g, 138.4 mmol), and 30 mL of 1,2-dichlorobenzene were added. The mixture was stirred at 180 °C for 24 h. Upon completion of the reaction, the mixture was cooled to room temperature, and the solvent was removed under a vacuum. The resulting compound was recrystallized from ethanol, yielding a white solid (2.36 g, 6.41 mmol) with a yield of 28%. ^1^H NMR (400 MHz, DMSO-*d*_6_, δ): 10.85 (s, 2H), 8.62 (s, 1H), 7.99 (d, *J* = 8.2 Hz, 2H), 7.35 (d, *J* = 1.7 Hz, 2H), 7.29 (d, *J* = 0.9 Hz, 1H), 7.17 (dd, *J* = 8.2, 1.7 Hz, 2H), 1.35 (s, 18H).

#### 2.2.5. Synthesis of 1,5-Dibromo-2,4-dinitrobenzene, (**5**)

In a 100 mL round-bottom flask, (4-fluorophenyl)boronic acid (0.16 g, 1.17 mmol), 2-chloro-4,6-diphenyl-1,3,5-triazine (0.3 g, 1.12 mmol), and 30 mL of anhydrous tetrahydrofuran (THF) were added and dissolved. Following dissolution, Pd(PPh_3_)_4_ (0.065 g, 0.056 mmol) and 2 mL of 2 M K_2_CO_3_ aqueous solution were introduced. The mixture was heated to 70 °C under a nitrogen atmosphere and stirred for 5 h. After the reaction was complete, the mixture was cooled to room temperature and quenched with deionized water. The resulting mixture was extracted with dichloromethane (MC) and deionized water. The organic layer was dried over anhydrous MgSO_4_ and filtered. The residue was purified using silica gel column chromatography using a 1:1 mixture of dichloromethane and n-hexane as the eluent, yielding a white solid (0.29 g, 0.886 mmol) with a yield of 80%. ^1^H NMR (400 MHz, Chloroform-*d*, δ): 7.86 (d, *J* = 7.6 Hz, 2H), 7.59 (dt, *J* = 8.2, 1.5 Hz, 4H), 7.46–7.41 (m, 4H), 7.38–7.33 (m, 2H), 1.35 (s, 12H)

#### 2.2.6. Synthesis of 1,5-Dibromo-2,4-dinitrobenzene, (**6**)

In a 100 mL round-bottom flask, 3-dibromo-5-chloro-2-fluorobenzene (0.3 g, 1.04 mmol) and phenylboronic acid (0.38 g, 3.12 mmol) were dissolved in 25 mL of anhydrous THF. Upon dissolution, Pd(PPh_3_)_4_ (0.12 g, 0.104 mmol) and 0.5 mL of 2 M K_2_CO_3_ aqueous solution were added. The reaction mixture was heated to 70 °C under a nitrogen atmosphere and stirred for 16 h. After the reaction was complete, the mixture was cooled to room temperature and quenched with deionized water. The resulting mixture was extracted using EA and deionized water. The organic layer was dried over anhydrous MgSO_4_ and filtered. The residue was purified using silica gel column chromatography using a 1:9 mixture of MC and n-hexane as the eluent, yielding a white solid (0.217 g, 0.769 mmol) with a yield of 74%. ^1^H NMR (400 MHz, Chloroform-*d*, δ): 7.57–7.52 (m, 4H), 7.46 (tt, *J* = 6.5, 1.1 Hz, 4H), 7.43–7.39 (m, 2H), 7.38 (d, *J* = 6.2 Hz, 2H).

#### 2.2.7. Synthesis of 1,5-Dibromo-2,4-dinitrobenzene, (**7**)

In a 100 mL round-bottom flask, compound (**6**) (1.00 g, 3.54 mmol), bis(pinacolato)diboron (1.34 g, 5.31 mmol), potassium acetate (0.87 g, 8.84 mmol), tri-cyclohexylphosphine (0.0990 g, 0.354 mmol), and tris(dibenzylideneacetone)dipalladium(0) (Pd_2_(dba)_3_) (0.323 g, 0.353 mmol) were added and dissolved in 50 mL of anhydrous 1,4-dioxane. The reaction mixture was refluxed under a nitrogen atmosphere for 24 h. Upon completion, the mixture was filtered and diluted with toluene, then washed with water. The organic layer was dried over anhydrous MgSO_4_. The dried product was purified using silica gel column chromatography using a 2:3 mixture of MC and n-hexane as the eluent, yielding a white solid (1.19 g, 3.19 mmol) with a yield of 87%. ^1^H NMR (400 MHz, Chloroform-*d*, δ): 7.86 (d, *J* = 7.6 Hz, 2H), 7.59 (dt, *J* = 8.2, 1.5 Hz, 4H), 7.46–7.41 (m, 4H), 7.38–7.33 (m, 2H), 1.35 (s, 12H).

#### 2.2.8. Synthesis of 1,5-Dibromo-2,4-dinitrobenzene, (**8**)

In a 100 mL round-bottom flask, compound (**7**) (0.5 g, 1.34 mmol) and 2-chloro-4,6-diphenyl-1,3,5-triazine (0.3 g, 1.12 mmol) were dissolved in 30 mL of anhydrous THF. After dissolution, Pd(PPh_3_)_4_ (0.065 g, 0.056 mmol) and 2 mL of 2 M K_2_CO_3_ aqueous solution were added. The reaction mixture was heated to 70 °C under a nitrogen atmosphere and stirred for 5 h. Upon completion, the mixture was cooled to room temperature and quenched with deionized water. The resulting mixture was extracted using MC and deionized water. The organic layer was dried over anhydrous MgSO_4_ and filtered. The residue was purified using silica gel column chromatography using a 1:1 mixture of dichloromethane and n-hexane as the eluent, yielding a white solid (0.35 g, 0.730 mmol) with a yield of 65%. ^1^H NMR (400 MHz, Chloroform-*d*, δ): 8.84 (d, *J* = 7.0 Hz, 2H), 8.78–8.72 (m, 4H), 7.72 (dq, *J* = 6.2, 1.5 Hz, 4H), 7.62–7.52 (m, 10H), 7.48–7.44 (m, 2H).

#### 2.2.9. Synthesis of 1,5-Dibromo-2,4-dinitrobenzene, 2TRZ-P-ICz

In a 100 mL round-bottom flask, compound (**4**) (0.2 g, 0.54 mmol) and cesium carbonate (Cs_2_CO_3_) (0.707 g, 2.17 mmol) were dissolved in 20 mL of N,N-dimethylformamide (DMF). A solution of compound (**5**) (0.389 g, 1.19 mmol) in 5 mL of DMF was added dropwise. Following the addition, the mixture was heated to 160 °C under a nitrogen atmosphere and stirred for 24 h. Upon completion of the reaction, the mixture was cooled to room temperature and quenched with deionized water. The resulting mixture was extracted using MC and deionized water. The organic layer was dried over anhydrous MgSO_4_ and filtered. The residue was purified using silica gel column chromatography using a 1:5 mixture of MC and n-hexane as the eluent, followed by recrystallization from a dichloromethane/methanol mixture, yielding a white solid (0.45 g, 0.464 mmol) with a yield of 36%. ^1^H NMR (400 MHz, Chloroform-*d*, δ): 9.02–8.97 (m, 4H), 8.79 (s, 1H), 8.76–8.72 (m, 8H), 8.19 (d, *J* = 8.2 Hz, 2H), 7.85–7.80 (m, 4H), 7.57–7.49 (m, 15H), 7.43 (dd, *J* = 8.2, 1.6 Hz, 2H), 1.40 (s, 18H); ^13^C NMR (101 MHz, Chloroform-*d,* δ): 171.75, 170.93, 149.21, 142.13, 141.54, 141.02, 136.13, 134.82, 132.62, 130.91, 129.13, 129.02, 128.69, 128.32, 126.88, 125.40, 122.01, 119.34, 118.38, 111.26, 106.25, 35.34, 31.92. HRMS (FAB-MS, m/z): [M+] calc’d for C_68_H_54_N_8_, 983.2360; found, 982.4476. Elemental analysis calculated (%) for C_68_H_54_N_8_: C, 83.07; H, 5.54; N, 11.40; found: C 83.68, H 5.79, N 10.35.

#### 2.2.10. Synthesis of 1,5-Dibromo-2,4-dinitrobenzene, 2TRZ-TP-ICz

In a 100 mL round-bottom flask, compound (**4**) (0.2 g, 0.54 mmol) and Cs_2_CO_3_ (0.707 g, 2.17 mmol) were dissolved in 20 mL of DMF. A solution of compound (**8**) (0.546 g, 1.14 mmol) in 5 mL of DMF was added dropwise. After the addition, the mixture was heated to 160 °C under a nitrogen atmosphere and stirred for 24 h. Upon completion of the reaction, the mixture was cooled to room temperature and quenched with deionized water. The resulting mixture was extracted using MC and deionized water. The organic layer was dried over anhydrous MgSO_4_ and filtered. The residue was purified using silica gel column chromatography using a 1:3 mixture of MC and n-hexane as the eluent. The purified product was then recrystallized from a dichloromethane/methanol mixture, yielding a yellow solid (0.245 g, 0.191 mmol) with a yield of 34%. ^1^H NMR (400 MHz, Chloroform-*d,* δ): 8.99 (s, 4H), 8.75–8.72 (m, 8H), 8.31 (d, *J* = 0.8 Hz, 1H), 7.79 (d, *J* = 8.2 Hz, 2H), 7.57–7.49 (m, 12H), 7.08 (dd, *J* = 8.2, 1.6 Hz, 2H), 6.97–6.93 (m, 4H), 6.88–6.86 (m, 16H), 6.79 (dd, *J* = 1.7, 0.6 Hz, 2H), 6.34 (d, *J* = 0.7 Hz, 1H), 1.22 (s, 18H); ^13^C NMR (101 MHz, Chloroform-*d,* δ): 171.94, 171.17, 147.86, 142.85, 140.96, 140.53, 138.99, 136.55, 136.24, 136.06, 132.74, 131.17, 129.11, 128.75, 128.19, 127.24, 121.17, 118.38, 117.76, 117.04, 110.33, 106.63, 34.99, 31.77; HRMS (FAB-MS, m/z): [M+] calculated for C_92_H_70_N_8_, 1287.6280; found, 1286.5725. Elemental analysis calculated (%) for C_92_H_70_N_8_: C, 85.82; H, 5.48; N, 8.70; found: C 84.95, H 5.60, N 8.21.

### 2.3. Lippert–Mataga Model

The Lippert–Mataga model can explain the relationship between the excited state’s dipole moment and solvent polarity. Based on the relevant dipole moment of the solvent, the dipole moment of the excited state can be calculated using the following equation [18,19].

$$\Delta \nu ={\bar{\nu }}_{a}-{\bar{\nu }}_{f}=\frac{2{\left(\mu_{E}-\mu_{G}\right)}^{2}}{hca^{3}}f(\varepsilon ,n)$$

Δ*ν* represents the Stokes shift, while ${\bar{\nu }}_{a}$ and ${\bar{\nu }}_{f}$ denote the wavenumbers of absorption and fluorescence emission, respectively. The term *f* refers to the orientational polarizability of the solvent, *μ_E_* is the dipole moment of the excited state, *μ_G_* is the dipole moment of the ground state, *a* is the Onsager cavity radius, and *ε* and *n* are the solvent’s dielectric constant and refractive index, respectively.

## 3. Results and Discussion

### 3.1. Molecular Design, Synthesis, and Characterization

**Scheme 1:**
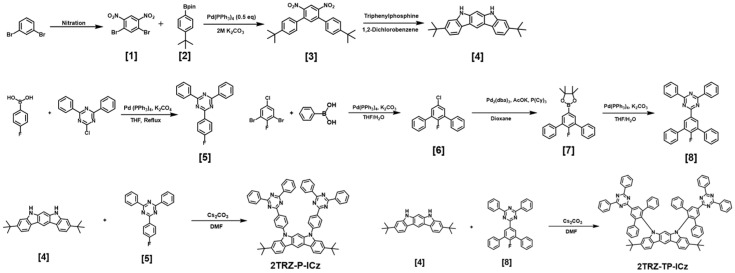
Synthetic routes of the synthesized compounds.

**Scheme 2:**
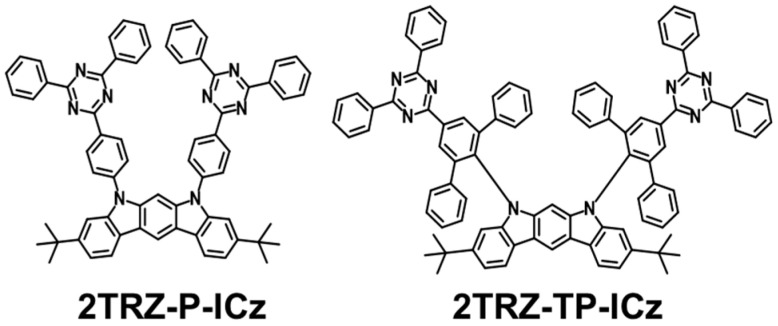
Chemical structures of newly synthesized host materials: 2TRZ-P-ICz and 2TRZ-TP-ICz.

In this study, a new luminescent material with bipolar characteristics was designed using ICz as the electron-donating moiety and TRZ as the electron-withdrawing moiety. ICz, although structurally similar to carbazole, exhibits a relatively stronger electron-donating effect, superior carrier mobility, and high rigidity [20,21,22]. However, due to the planar molecular structure of ICz, a reduction in intermolecular distance in the film state can enhance intermolecular π–π interactions, leading to aggregation. This aggregation can cause quenching effects, thereby decreasing the luminescence efficiency. To prevent this, a t-butyl group was substituted onto the ICz, and a phenyl group or TP group was introduced as a linker between the donor and acceptor moieties. Consequently, two types of compounds with different linker structures, 2TRZ-P-ICz and 2TRZ-TP-ICz, were synthesized. The TP linker, being bulky, induces intramolecular steric hindrance and increases the dihedral angles, effectively suppressing intermolecular interactions. Additionally, the increased dihedral angle is expected to reduce the energy level difference (ΔE_ST_) between the S_1_ and T_1_ states, thereby enhancing the luminescence characteristics through improved reverse intersystem crossing (RISC). The synthetic routes and molecular structures of 2TRZ-P-ICz and 2TRZ-TP-ICz are presented in Scheme 1 and Scheme 2. The final compounds were synthesized using reactions such as nitration, Miyaura borylation, Suzuki coupling, Cadogan cyclization, and substitution of aromatic halides with nitrogen nucleophiles. All compounds were purified using recrystallization and column chromatography. The chemical structures of the synthesized final compounds were characterized using ^1^H NMR, ^13^C NMR, mass spectrometry, and elemental analysis (Figures S1–S12).

### 3.2. Photophysical Properties

**Figure 1:**
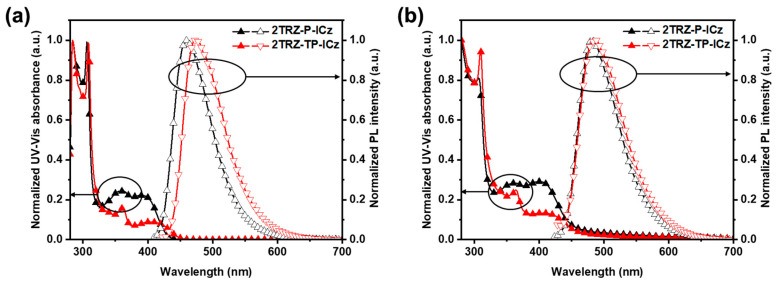
Normalized UV–visible absorption and PL spectra of newly synthesized compounds in (**a**) the solution state (toluene, 1 × 10^−5^ M) and (**b**) as a vacuum-deposited film (thickness: 50 nm).

To evaluate the photophysical properties of the newly synthesized compounds 2TRZ-P-ICz and 2TRZ-TP-ICz, their Ultraviolet–visible (UV–vis) absorption and photoluminescence (PL) spectra were measured in toluene solution and in the vacuum-deposited film state, as shown in Figure 1 and Figure S13. The photophysical properties of these new compounds are summarized in Table 1. Both compounds exhibited strong absorption peaks in the range of approximately 285–310 nm, corresponding to the localized π–π* transitions of the aromatic rings containing the donor and acceptor moieties. Weak absorption peaks in the range of 320–400 nm were attributed to the delocalized n–π* transitions of indolocarbazole. Additionally, a broad absorption band in the range of 325–400 nm was observed for all compounds, which can be attributed to intramolecular charge transfer (ICT) between the donor indolocarbazole and the acceptor triazine. In the solution state, the PL maximum wavelengths (λ_PL_) of 2TRZ-P-ICz and 2TRZ-TP-ICz were 460 nm and 473 nm, respectively. The 2TRZ-TP-ICz, with the introduction of the TP linker group, exhibited a red-shifted emission compared to 2TRZ-P-ICz, corresponding to a longer conjugation length. In the film state, the λ_PL_ values of 2TRZ-P-ICz and 2TRZ-TP-ICz were 480 nm and 488 nm, showing red-shifts of 20 nm and 15 nm compared to their solution state values, respectively. The newly synthesized compounds exhibit a red-shift in emission wavelength in the film state because the intermolecular distance decreases, leading to increased intermolecular π–π interactions. Particularly for 2TRZ-P-ICz, the larger dipole moment compared to 2TRZ-TP-ICz results in a closer intermolecular distance in the film state, allowing the dipole interactions to be more effective, thereby causing a greater red-shift in the emission wavelength. In the film state, the closer intermolecular distances generally enhance intermolecular interactions, which can lead to various transition states that increase non-radiative decay. This results in a decrease in luminescence efficiency, a red-shift in the λ_PL_, and a broadening of the full-width at half-maximum (FWHM) of the PL spectrum. However, for 2TRZ-TP-ICz, the bulky linker helps to suppress intermolecular packing, resulting in a smaller shift in wavelength compared to 2TRZ-P-ICz. In solution, the FWHM values for 2TRZ-P-ICz and 2TRZ-TP-ICz were 67 nm and 71 nm. In the film state, the FWHM values increased to 71 nm and 77 nm, showing only a modest increase.

**Figure 2:**
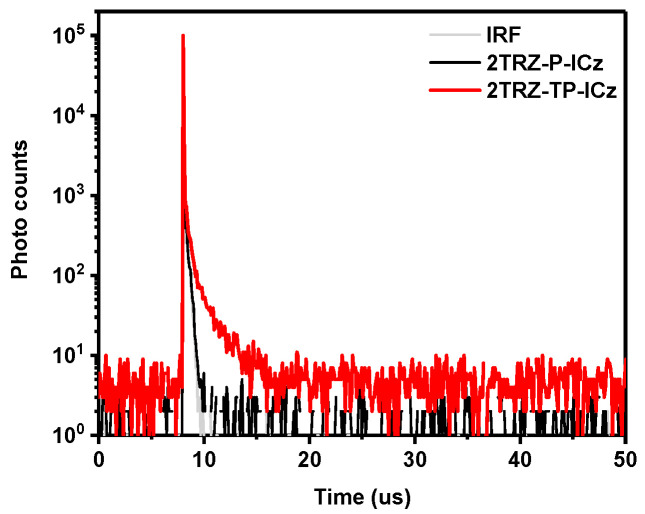
Transient photoluminescence decay spectra of the neat films (IRF: instruments response function, Range: 50 μs).

To further investigate this, delayed fluorescence in the neat film of newly synthesized host materials was confirmed based on transient PL (TRPL) measurements, as shown in Figure 2 and Table S1. The prompt fluorescence lifetimes (τ_p_) of 2TRZ-P-ICz and 2TRZ-TP-ICz were 2.69 ns and 22.20 ns, and their delayed fluorescence lifetimes (τ_d_) were 0.188 μs and 2.080 μs, respectively. Based on the delayed lifetime values, the calculated rate constants for reverse intersystem crossing (k_RISC_) in the neat films of 2TRZ-P-ICz and 2TRZ-TP-ICz were 4.33 × 10^2^/s and 3.27 × 10^3^/s [23]. Therefore, 2TRZ-TP-ICz exhibited superior TADF characteristics, as indicated by its relatively high k_RISC_ value. For 2TRZ-P-ICz, the transient PL spectrum did not show evidence of delayed fluorescence, and the value of τ_d_ was 0.188 μs. Even if considered up to a maximum of 0.2 μs, it remained below 1 μs, indicating that it did not exhibit TADF characteristics. The incorporation of the TP linker effectively suppressed intramolecular rotation and vibration, achieving a small ΔE_ST_, which is expected to result in higher device efficiency when applied as an emitter compared to 2TRZ-P-ICz. The absolute PLQY values were measured in both solution and neat film states. In solution, the PLQY values for 2TRZ-P-Cz and 2TRZ-TP-Cz were 57.1% and 43.6%, respectively. In the neat film state, the PLQY values were 33.8% and 32%. As previously explained, the decrease in PLQY in the film state compared to the solution state is attributed to emission quenching caused by intermolecular aggregation in the solid state, which leads to a reduction in efficiency [22].

The HOMO values were determined based on the ultraviolet photoelectron spectra (Riken–Keiki, AC-2), and the optical band gaps were determined by measuring the absorption edges from plots of (hν) versus (αhν)^2^, where α, h, and ν represent the absorbance, Planck’s constant, and the frequency of light, respectively. The LUMO level was obtained using the HOMO level measured as described above and the optical bandgap obtained through theoretical calculation. The HOMO levels of 2TRZ-P-ICz and 2TRZ-TP-ICz were −5.50 eV and −5.59 eV, respectively, with band gaps of 2.80 eV and 2.75 eV, as shown in Table 1 and Figure S14. Due to the extended conjugation induced by the TP linker, 2TRZ-TP-ICz exhibits a slightly smaller band gap compared to 2TRZ-P-ICz.

**Figure 3:**
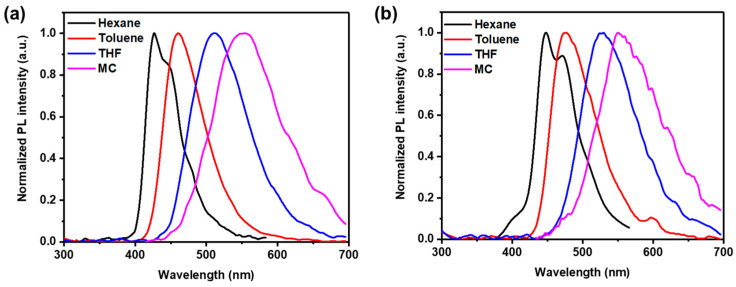
PL spectra according to solvents with different dielectric constants of (**a**) 2TRZ-P-ICz and (**b**) 2TRZ-TP-ICz.

To investigate the bipolar properties of the newly synthesized emitters, solvatochromism experiments were conducted using solvents with varying polarities (dielectric constants: n-hexane (1.90) < toluene (2.38) < tetrahydrofuran (7.58) < methyl chloride (8.93)), as shown in Figure 3. Both materials exhibited a red shift in the λ_PL_ as the polarity of the solvent increased. This behavior can be attributed to the stabilization of the excitation state with increasing solvent polarity, which decreases the band gap and results in a red shift of the PL wavelength. Furthermore, stronger solvent interactions contribute to a broader FWHM [24]. For 2TRZ-P-ICz, the λ_PL_ demonstrated a significant shift of 127 nm between hexane and methyl chloride, whereas 2TRZ-TP-ICz exhibited a shift of 81 nm. In the case of 2TRZ-P-Cz, the phenyl linker results in a relatively more linear structure compared to 2TRZ-TP-ICz, leading to stronger interactions with the solvent. This experiment confirms the bipolar properties of the synthesized materials. This behavior can be explained by the dipole moment values obtained from the Lippert–Mataga equation, which are 26.9 D for 2TRZ-P-ICz and 21.3 D for 2TRZ-TP-ICz, and the dipole moment values obtained from time-dependent DFT calculations, which are 3.07 D for 2TRZ-P-ICz and 2.33 D for 2TRZ-TP-ICz as shown in Figure S15 and Table S2.

The thermal properties of the synthesized host materials were evaluated using thermogravimetric analysis (TGA) and differential scanning calorimetry (DSC) experiments, and the results are summarized in Table 1. As shown in Figure S16, the T_d_ corresponding to 5% weight loss for 2TRZ-P-ICz and 2TRZ-TP-ICz was 504 °C and 518 °C. 2TRZ-TP-ICz exhibited increased thermal stability due to the introduction of the bulky TP linker, which raised the molecular weight. Molecules with such high T_d_ values typically show significant resistance to the heat generated during OLED device operation, providing stable device performance [25]. However, the T_g_, T_m_, and T_c_ values of the newly synthesized compounds were not observed up to 350 °C (Figure S17).

### 3.3. Theoretical Calculation

**Figure 4:**
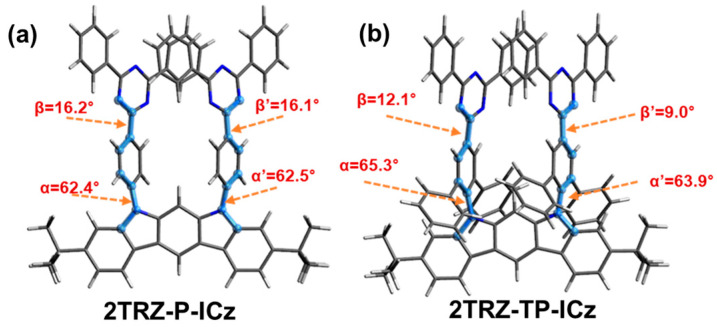
Optimized structures and dihedral angles calculated using B3LYP-D3/def2-TZVPP with ORCA: (**a**) 2TRZ-P-ICz and (**b**) 2TRZ-TP-ICz.

**Figure 5:**
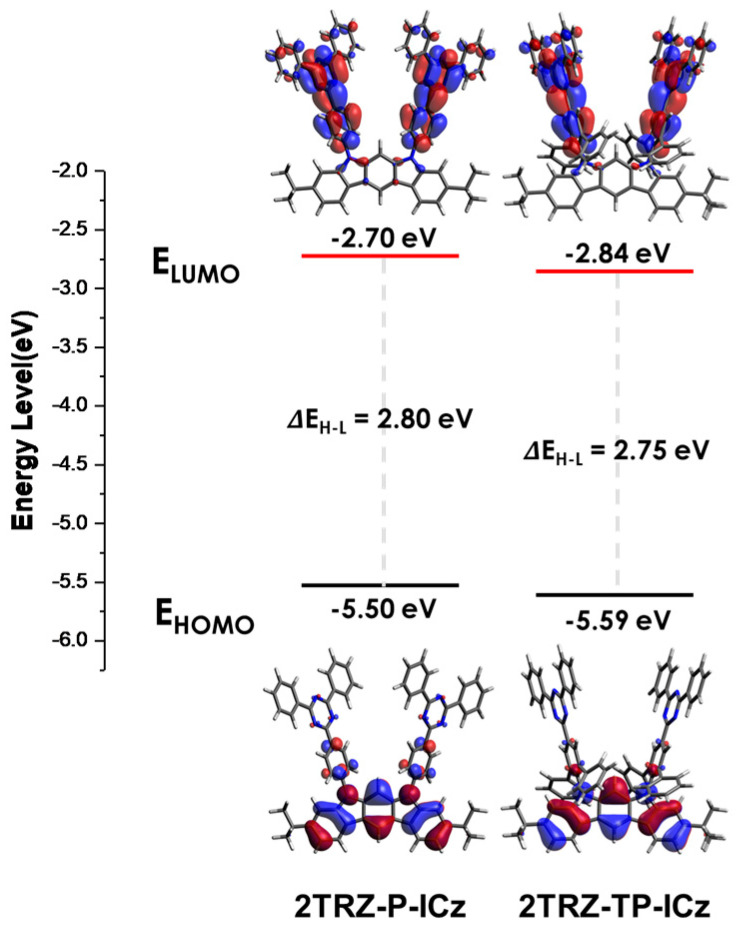
Electron density distributions and HOMO/LUMO energy levels.

To gain a detailed understanding of the molecular structures, optimized structures were calculated using the ORCA computational program at the B3LYP-D3/def2-TZVPP level, as shown in Figure 4. For 2TRZ-TP-Cz, the dihedral angles between the ICz and TP linker (α-α’) were 65.3° and 63.9°, respectively, while for 2TRZ-P-Cz, the dihedral angles between the ICz and phenyl linker (α-α’) were 62.4° and 62.5°. The dihedral angles between the linker and TRZ (β-β’) were 16.2° and 16.1° for 2TRZ-P-Cz and 12.1° and 9.0° for 2TRZ-TP-Cz, respectively. The electron density distributions of the highest occupied molecular orbital (HOMO) and lowest unoccupied molecular orbital (LUMO) for the new synthesized compounds were calculated using DFT (Figure 5). In a typical bipolar structure, the electron density of the LUMO is mainly located on the electron acceptor, while the electron density of the HOMO is primarily concentrated on the electron donor, resulting in a clear separation between the HOMO and LUMO [26,27]. In both materials, the LUMO electron density is observed in the electron acceptor region, triazine, and the linker, while the electron density of the HOMO is primarily located in the electron donor region, indolocarbazole. However, in 2TRZ-P-Cz, there is a slight presence of electron density in the electron acceptor triazine within the HOMO, indicating that 2TRZ-P-Cz is more linear compared to 2TRZ-TP-Cz. This observation is consistent with the results from the optimized structure calculations. Although the band gaps obtained from the molecular calculations for the HOMO and LUMO levels were 3.02 eV and 2.92 eV, which differed from the experimental values, the trends were consistent, as shown in Table S3.

### 3.4. Electroluminescence (EL) Properties

**Figure 6:**
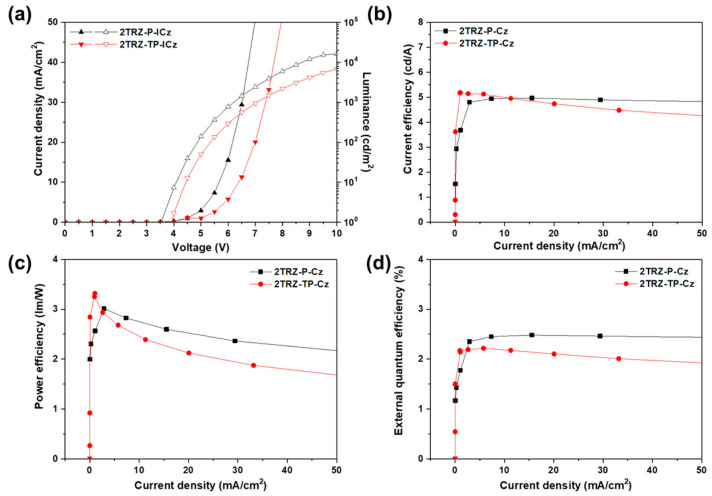
EL performance of non-doped OLED devices: (**a**) current density (J)–voltage (V)–luminance (L) characteristics, (**b**) CE vs. J curves, (**c**) PE vs. J curves, (**d**) EQE vs. J curves (device configuration: ITO/2TNATA (60 nm)/NPB (15 nm)/EML (30 nm)/TPBi (35 nm)/LiF (1 nm)/Al (200 nm)).

**Figure 7:**
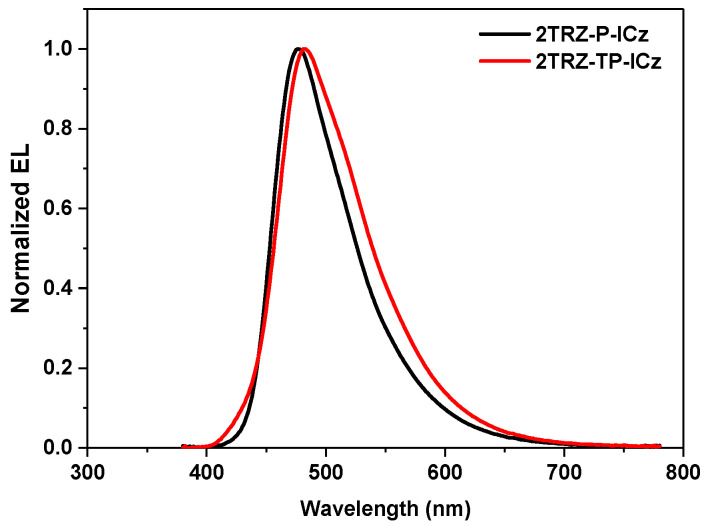
EL spectra of non-doped devices.

To evaluate the EL performance of the newly synthesized materials, non-doped devices were fabricated using 2TRZ-P-Cz and 2TRZ-TP-ICz as the emitting layers (EML). The device structure was as follows: ITO/4,4′,4″-Tris(N-(naphthalen-2-yl)-N-phenyl-amino)-triphenylamine (2TNATA) (60 nm)/N,N′-di(1-naphthyl)-N,N′-diphenyl-(1,1′-biphenyl)-4,4′-diamine (NPB) (15 nm)/EML (30 nm)/1,3,5-Tris(1-phenyl-1H-benzimidazol-2-yl)benzene (TPBi) (35 nm)/lithium fluoride (LiF) (1 nm)/aluminium (Al) (200 nm). In this study, 2TNATA was used as the hole injection layer (HIL), and NPB served as the hole-transporting layer (HTL). TPBI served as the electron-transporting layer (ETL), and LiF was applied as the electron injection layer (EIL). ITO and Al were used as the anode and cathode, respectively. Figure 6 illustrates the current density (J)–voltage (V)–luminance (L), current efficiency (CE)–J, power efficiency (PE)–J, and EQE–J curves of the OLED devices. The device lifetime is not determined solely by a single luminescent material but is influenced by many other factors. Therefore, a comparison of device lifetimes was not included in this study. The EL performance data of the devices are summarized in Figure 6 and Figure 7 and Table 2. At a current density of 10 mA/cm^2^, the operating voltages for the 2TRZ-P-Cz and 2TRZ-TP-ICz devices were 5.6 V and 6.4 V, respectively, with the 2TRZ-TP-ICz device exhibiting a 0.8 V higher operating voltage. Additionally, the 2TRZ-TP-ICz device exhibited a maximum current efficiency (CE_max_) of 5.17 cd/A and a maximum external quantum efficiency (EQE_max_) of 2.21%. Analysis of the EL characteristics shows that 2TRZ-P-ICz has an EL maximum (EL_max_) at 477 nm with a CIE coordinate of (0.181, 0.304), indicating a blue shift compared to 2TRZ-TP-ICz, which has an EL_max_ at 482 nm and a CIE coordinate of (0.206, 0.363). The EL_max_ of the non-doped devices fabricated in this study was similar to the λ_PL_ of the materials in the film state.

To evaluate the bipolar characteristics of the newly synthesized host materials, single-carrier devices were fabricated, and the space-charge-limited current (SCLC) method was used to investigate charge transport ability, as shown in Figure S18. For this purpose, hole-only devices (HODs) were fabricated with the structure ITO/molybdenum trioxide (MoO_3_) (1 nm)/EML (50 nm)/MoO_3_ (10 nm)/Al (100 nm) to measure hole carrier mobility, while electron-only devices (EODs) were fabricated with the structure ITO/TmPyPb (40 nm)/EML (50 nm)/TmPyPb (40 nm)/LiF (1 nm)/Al (100 nm) to measure electron carrier mobility. The hole and electron mobility of host materials was estimated using the modified Mott−Gurney space-charge-limited current (SCLC) method according to the following equation [28,29]:

(1) $$J=\frac{9}{8}\mu \varepsilon \varepsilon_{0}\frac{V^{3}}{d^{3}}$$

where ε is the relative dielectric constant of organic semiconductors, $\varepsilon_{0}$ is the vacuum permittivity, V is $V_{appl}-V_{bi}$ (where $V_{appl}$ is the applied voltage and $V_{bi}$ is the work function difference between the two electrodes), and *d* is the thickness of the active layer.

In the HOD, the measured hole mobility values were 3.43 × 10^−3^ cm^2^/Vs for 2TRZ-P-ICz and 2.16 × 10^−3^ cm^2^/Vs for 2TRZ-TP-ICz at 1V. In the EOD, the measured electron mobility values were 4.41 × 10^−9^ cm^2^/Vs for 2TRZ-P-ICz and 9.13 × 10^−9^ cm^2^/Vs for 2TRZ-TP-ICz at 1V. The carrier mobility of the molecules is influenced by the inductive effects of the substituents within the molecule and the molecular structural arrangement [30,31]. The dihedral angle between ICz and the linker in 2TRZ-P-Cz is similar to that in 2TRZ-TP-ICz, but the hole mobility is increased by 1.58 times. 2TRZ-TP-Cz, which has a TP linker, exhibits a relatively lower hole mobility compared to 2TRZ-P-Cz due to the steric effect of the TP linker, increasing the spatial distance between molecules. However, the dihedral angle between the linker and TRZ in 2TRZ-TP-Cz is relatively smaller than in 2TRZ-P-Cz, which reduces the spatial distance between molecules and consequently increases the electron mobility by 2.07 times compared to 2TRZ-P-Cz.

**Figure 8:**
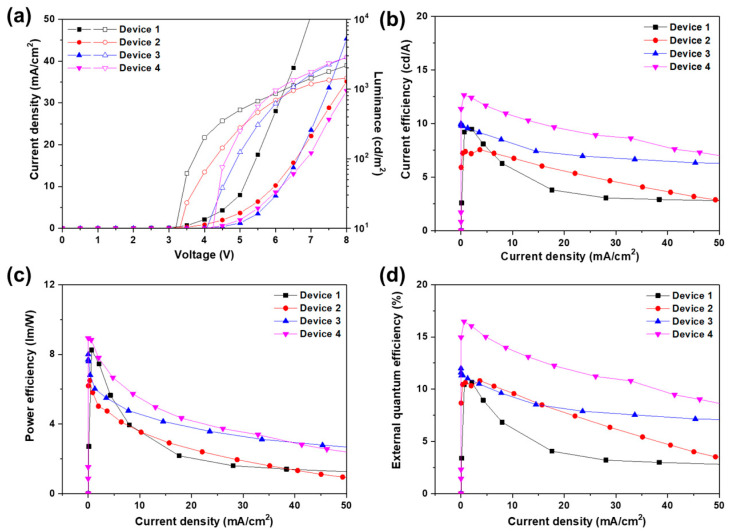
EL performance of doped OLED devices: (**a**) current density (J)–voltage (V)–luminance (L) characteristics, (**b**) CE vs. J curves, (**c**) PE vs. J curves, (**d**) EQE vs. J curves (device configuration: ITO/HAT-CN (5 nm)/TAPC (30 nm)/TCTA (10 nm)/Host: X wt% Ir(piq)_2_(acac) (20 nm)/TmPyPB (30 nm)/LiF (1 nm)/Al (200 nm)).

**Figure 9:**
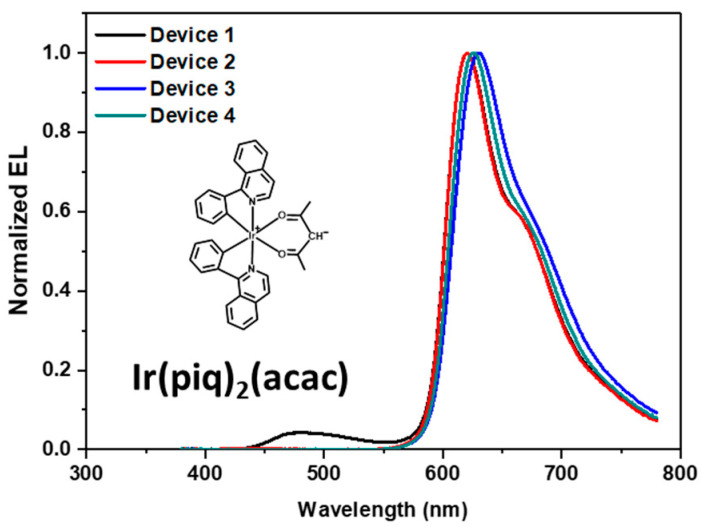
EL spectra of doped devices at 10 mA/cm^2^.

Additionally, 2TRZ-P-ICz and 2TRZ-TP-ICz were used as phosphorescent red hosts to utilize their bipolar characteristics and TADF properties. The related properties were evaluated and summarized in Figure 8, Figure 9 and Figure S19, and Table 3. The three newly synthesized materials were used as red host materials in the fabrication of doped devices with the following structure: ITO/1,4,5,8,9,11-hexaazatriphenylenehexacarbonitrile (HAT-CN) (10 nm)/1,1-bis[(di-4-tolylamino)phenyl]cyclohexane (TAPC) (30 nm)/tris(4-carbazoyl-9-ylphenyl)amine (TCTA) (10 nm)/host: 4 or 10 wt% bis(1-phenylisoquinoline)(acetylacetonate)iridium(III) (Ir(piq)_2_(acac)) (20 nm)/1,3,5-tris(3-pyridyl-3-phenyl)benzene (TmPyPb) (40 nm)/LiF (1 nm)/Al (200 nm). In this device, HAT-CN was employed as the hole injection layer, TAPC served as the hole-transporting layer, TCTA acted as the exciton blocking layer (EBL), TmPyPb functioned as both the electron-transporting layer (ETL) and the hole-blocking layer (HBL), and LiF and Al were used as the electron-injecting material and the cathode, respectively. Devices 3 and 4 employed 2TRZ-TP-ICz, while Devices 1 and 2 utilized 2TRZ-P-ICz as the host material. The dopant concentrations were set at 4 wt% and 10 wt%. To evaluate the performance of the red phosphorescent host, we first confirmed efficient energy transfer between the host material and Ir(piq)_2_(acac) prior to device fabrication. This was achieved by examining the overlap between the PL spectra of the host materials in the film state and the UV absorption spectra of Ir(piq)_2_(acac) in the solution state, as shown in Figure S20. The observed overlap indicates that Förster energy transfer from the host to the dopant occurred efficiently. The T₁ levels of 2TRZ-P-ICz and 2TRZ-TP-ICz were 2.72 eV and 2.75 eV, respectively, which were higher than the T₁ level of the dopant Ir(piq)_2_(acac), which was 1.98 eV. Therefore, the Dexter energy transfer was also expected to be efficient. Additionally, to verify the efficiency of energy transfer from the host materials to the dopant, Stern–Volmer experiments were conducted and summarized in Figure S20 and Table S4. The quenching rate constants (k_q_) for 2TRZ-P-ICz and 2TRZ-TP-ICz are 1.44 × 10^7^ and 1.82 × 10^7^/s. When 2TRZ-TP-ICz is used as the host, energy transfer to the dopant occurs more efficiently and rapidly than with 2TRZ-P-ICz.

In the case of phosphorescence, a higher dopant concentration is required compared to fluorescent dopants due to Dexter energy transfer. Typically, the dopant concentration for phosphorescence is used in the range of 4 to 10 wt%, whereas for fluorescence, it is used in the range of 1 to 5 wt%. For our device experiment, we selected and tested the lowest concentration of 4 wt% and the highest concentration of 10 wt%. For devices with a doping concentration of 4 wt%, Device 1 and Device 3 exhibited CE values of 5.77 and 8.10 cd/A, respectively, and EQE values of 6.27% and 9.12%. Although Device 3, using 2TRZ-TP-ICz as the host, demonstrated a higher efficiency, the EL spectrum of Device 1 revealed a peak at 470 nm corresponding to the host 2TRZ-P-ICz. This indicates insufficient energy transfer from the host to the dopant. To address this issue, the doping concentration was increased to 10 wt%. Devices 2 and 4, with this higher doping concentration, exhibited similar operating voltages of approximately 6 V. However, the CE improved to 6.81 and 9.92 cd/A, and the EQE increased to 9.58% and 13.7%. Device 4, using 2TRZ-TP-ICz as the host, exhibited an overall efficiency approximately 1.4 times higher than Device 2, which used 2TRZ-P-ICz as the host. The Jinhai Huang group reported a maximum EQE of 13.82% for red phosphorescent devices using a bipolar host based on carbazole and dioxy[2,3-b] pyrazine, specifically 7-(9-phenyl-9H-[3,9′-bicarbazole]-3′-yl) benzo[1,4,5,6] dioxy[2,3-b] pyrazine (b-CzDp). In comparison, Device 4 demonstrated an improved performance with a maximum EQE of 16%, representing a 1.15-fold enhancement [32]. In the case of 2TRZ-P-ICz, it exhibits a lower k_q_ value compared to 2TRZ-TP-ICz, indicating that energy transfer from the host to the dopant is not efficiently occurring. Additionally, the large ΔE_ST_ and slow k_RISC_ of 2TRZ-P-ICz contribute to its relatively lower efficiency. On the other hand, 2TRZ-TP-ICz achieved a higher device efficiency due to its higher k_q_ value, smaller ΔE_ST_, and faster k_RISC_ compared to 2TRZ-P-ICz. Upon examining the EL spectrum, the EL peak of Ir(piq)_2_(acac) was observed in both devices. The CIE coordinates of Devices 2 and 4 were (0.6372, 0.324) and (0.679, 0.319), indicating similar chromaticity. This suggests that, unlike Devices 1 and 3, efficient energy transfer from the host to the dopant occurred. However, with increased doping concentrations, aggregation caused the FWHM to broaden in comparison to Devices 1 and 3.

## 4. Conclusions

New red host materials were designed and synthesized using ICz as an electron-donating group, TRZ as an electron-withdrawing group, and a phenyl linker with either a phenyl or TP group. Compared to 2TRZ-TP-ICz, 2TRZ-P-ICz exhibited relatively stronger bipolar characteristics, as confirmed by solvatochromism experiments. 2TRZ-P-ICz demonstrated a relatively large ΔE_ST_ value of 0.21 eV and did not exhibit delayed fluorescence. In the case of 2TRZ-TP-ICz, although it exhibited relatively weaker bipolar characteristics, the introduction of the TP linker suppressed intramolecular rotation and vibration, resulting in a small ΔE_ST_ of 0.08 eV, which facilitated normal TADF properties. Evaluation of the new bipolar compounds as phosphorescent red hosts in doped devices revealed that, at a doping concentration of 4%, the energy transfer from the host to the dopant was insufficient in the 2TRZ-P-ICz host, whereas 2TRZ-TP-ICz facilitated efficient energy transfer. In experiments with 10wt% doped devices used to facilitate efficient energy transfer, 2TRZ-TP-ICz demonstrated a CE of 9.92 cd/A and an EQE of 13.7%. The high device efficiency was attributed to the small ΔE_ST_ and rapid k_RISC_ (3.47 × 10^3^/s). These results indicate that the introduction of a TP linker into bipolar molecules facilitates efficient energy transfer and represents a practical approach to achieving high-efficiency OLEDs using red phosphorescent host materials. Applying this method to emitters of other colors and red phosphorescent host materials could potentially lead to improved OLED characteristics with new emitters.

## Figures and Tables

**Table 1:** Photophysical and thermal properties of the synthesized materials.

	Solution ^a^		Film ^b^		Φ ^c^ [%]	HO MO ^d^ [eV]	LU MO [eV]	Band Gap [eV]	S_1_ ^e^ [eV]	T_1_ ^f^ [eV]	ΔE_ST_ ^g^ [eV]	T_d_ ^h^ [°C]
	λ_abs_ [nm]	λ_PL_ (FWHM) [nm]	λ_abs_ [nm]	λ_PL_ (FWHM) [nm]								
2TRZ-P-ICz	284, 306, 362, 390	460 (67)	306, 363, 400	480 (71)	57.1/33.8	−5.50	−2.70	2.80	2.93	2.72	0.21	504
2TRZ-TP-ICz	284, 306, 362, 403	473 (71)	309, 364, 404	488 (77)	43.6/32	−5.59	−2.84	2.75	2.83	2.75	0.08	518

^a^ Toluene solution, 1.0 × 10^−5^ M; ^b^ film thickness is 50 nm; ^c^ absolute photoluminescence quantum yield of solution/film state; ^d^ HOMO level was measured using ultraviolet photoelectron spectroscopy (Riken–Keiki, AC-2); ^e^ singlet energy calculated from onset point of the RTPL; ^f^ triplet energy calculated from onset point of the LTPL; ^g^ ΔE_ST_ was obtained based on the difference between singlet and triplet energies; ^h^ 5% weight loss.

**Table 2:** EL performance of non-doped device.

	Voltage ^a^ [V]	CE_max_ [cd/A]	PE_max_ [lm/W]	EQE_max_ [%]	CIE (x, y) ^a^	EL_max_ ^a^ [nm]
2TRZ-P-ICz	5.6	4.96	3.01	2.48	(0.181, 0.304)	477 (73)
2TRZ-TP-ICz	6.4	5.17	3.31	2.21	(0.206, 0.363)	482 (83)

^a^ at 10mA/cm^2^.

**Table 3:** EL performance of Ir(piq)_2_(acac)-doped OLED devices.

@10mA/cm^2^		Voltage [V]	CE [cd/A]	PE [lm/W]	EQE[%]	CIE (x, y)	EL_max_ (FWHM) [nm]
Device 1	2TRZ-P-ICz: 4wt% Ir(piq)_2_(acac)	5.10	5.77	3.57	6.27	(0.635, 0.327)	620 nm (78)
Device 2	2TRZ-P-ICz: 10wt% Ir(piq)_2_(acac)	6.00	6.81	3.57	9.58	(0.682, 0.316)	630 nm (81)
Device 3	2TRZ-TP-ICz: 4wt% Ir(piq)_2_(acac)	6.17	8.10	4.21	9.12	(0.672, 0.324)	620 nm (78)
Device 4	2TRZ-TP-ICz: 10wt% Ir(piq)_2_(acac)	6.15	9.92	5.50	13.7	(0.679, 0.319)	626 nm (78)

## Data Availability

The data are contained within the article and Supplementary Materials.

## Supplementary Materials

The following supporting information can be downloaded at: https://www.mdpi.com/article/10.3390/ma17174347/s1, Figure S1. ^1^H-NMR spectrum of compound (**1**) in chloroform-*d*; Figure S2. ^1^H-NMR spectrum of compound (**2**) in chloroform-*d*; Figure S3. ^1^H-NMR spectrum of compound (**3**) in chloroform-*d*; Figure S4. ^1^H-NMR spectrum of compound (**4**) in DMSO-*d_6_*; Figure S5. ^1^H-NMR spectrum of compound (**5**) in chloroform-*d*; Figure S6. ^1^H-NMR spectrum of compound (**6**) in chloroform-*d*; Figure S7. ^1^H-NMR spectrum of compound (**7**) in chloroform-*d*; Figure S8. ^1^H-NMR spectrum of compound (**8**) in chloroform-*d*; Figure S9. ^1^H-NMR spectrum of 2TRZ-P-ICz in chloroform-*d*; Figure S10. ^13^C-NMR spectrum of 2TRZ-P-ICz in chloroform-*d.* Figure S11. ^1^H-NMR spectrum of 2TRZ-TP-ICz in chloroform-*d.* Figure S12. ^13^C-NMR spectrum of 2TRZ-TP-ICz in chloroform-*d.* Figure S13. PL spectra acquired under room-temperature (RT) and low-temperature (LT) conditions for (a) 2TRZ-P-ICz and (b) 2TRZ-TP-ICz. Figure S14. Photoelectron yield spectra (AC2) of (a) 2TRZ-P-ICz and (b) 2TRZ-TP-ICz in neat films. Figure S15. Lippert–Mataga solvatochromic model of (a) 2TRZ-P-ICz and (b) 2TRZ-TP-ICz. Figure S16. Thermo-gravimetric analyzer (TGA) results of the synthesized compounds. Figure S17. Differential scanning calorimetry (DSC) curves of (a) 2TRZ-P-ICz and (b) 2TRZ-TP-ICz (heating rate: 10 °C/min, cooling rate: 10 °C/min). Figure S18. (a) Current density(J)–voltage(V) curves of the hole- and electron-only devices. Plot of (b) hole mobility and (c) electron mobility values as a function of E^1/2^. Figure S19. Band diagrams of the fabricated OLED devices: (a) non-doped device, (b) doped device. Figure S20. Spectral overlap between Ir(piq)_2_(acac) and newly synthesized host materials. Figure S21. Stern–Volmer plots: (a) 2TRZ-P-ICz and (b) 2TRZ-TP-ICz. Table S1. Rate constant for the synthesized materials (non-doped film) at room temperature. Table S2. Fitting parameters of Lippert–Mataga plots and Onsager radius (a), Δ*μ* (*μ_E_*–*μ_G_*) refers to the change in dipole moment. Table S3. HOMO and LUMO energy levels and band gaps of synthesized materials calculated using B3LYP-D3/def2-TZVPP with ORCA. Table S4. Rate constant of energy transfer between host and dopant based on Stern–Volmer equation.
