# Supplementary material for: New Bipolar Host Materials Based on Indolocarbazole for Red Phosphorescent OLEDs
Source: Materials (Basel). 2024 Sep 2;17(17):4347. doi: 10.3390/ma17174347 (PMC11396266; doi:10.3390/ma17174347)
Supplement: Supplementary file 1 [file materials-17-04347-s001.zip › materials-3176477-supplementary.pdf]

New bipolar host materials based on indolocarbazole for red phosphorescent OLEDs

Sunwoo Park, Hyukmin Kwon, Sangwook Park, Saeyoung Oh, Kiho Lee, Hayoon Lee, and Jongwook Park*

Integrated Engineering, Department of Chemical Engineering, Kyung Hee University, Gyeonggi, 17104, Korea

*E-mail: jongpark@khu.ac.kr

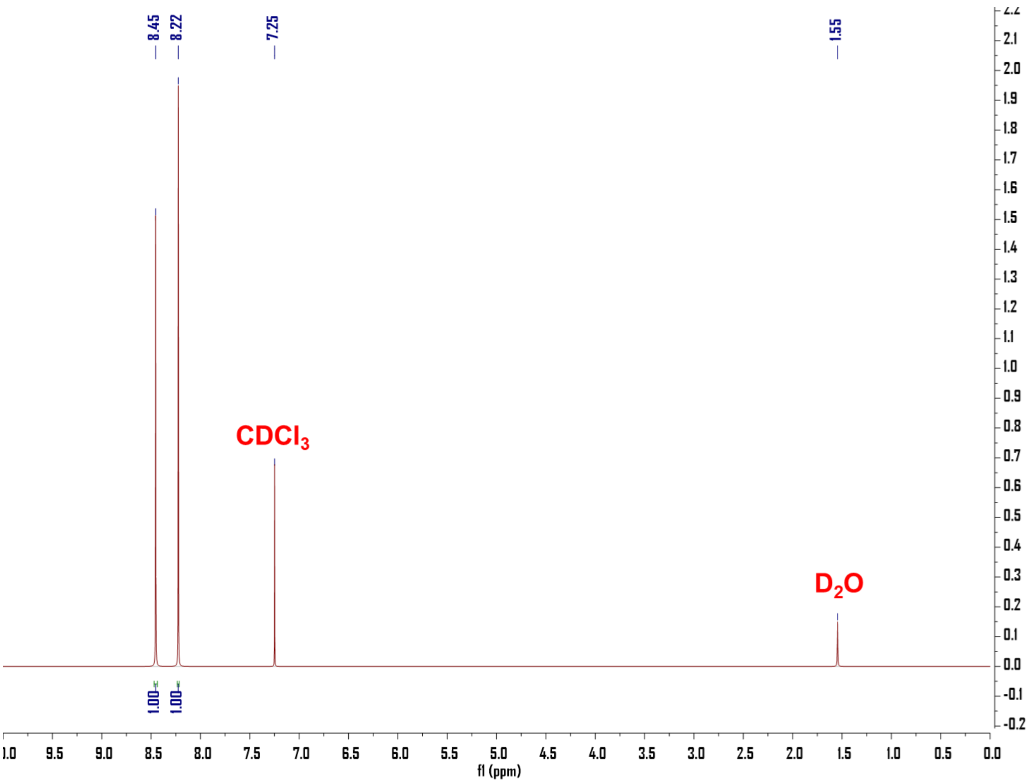

Figure S1. ^{1}H-NMR spectrum of compound (1) in Chloroform-*d*.

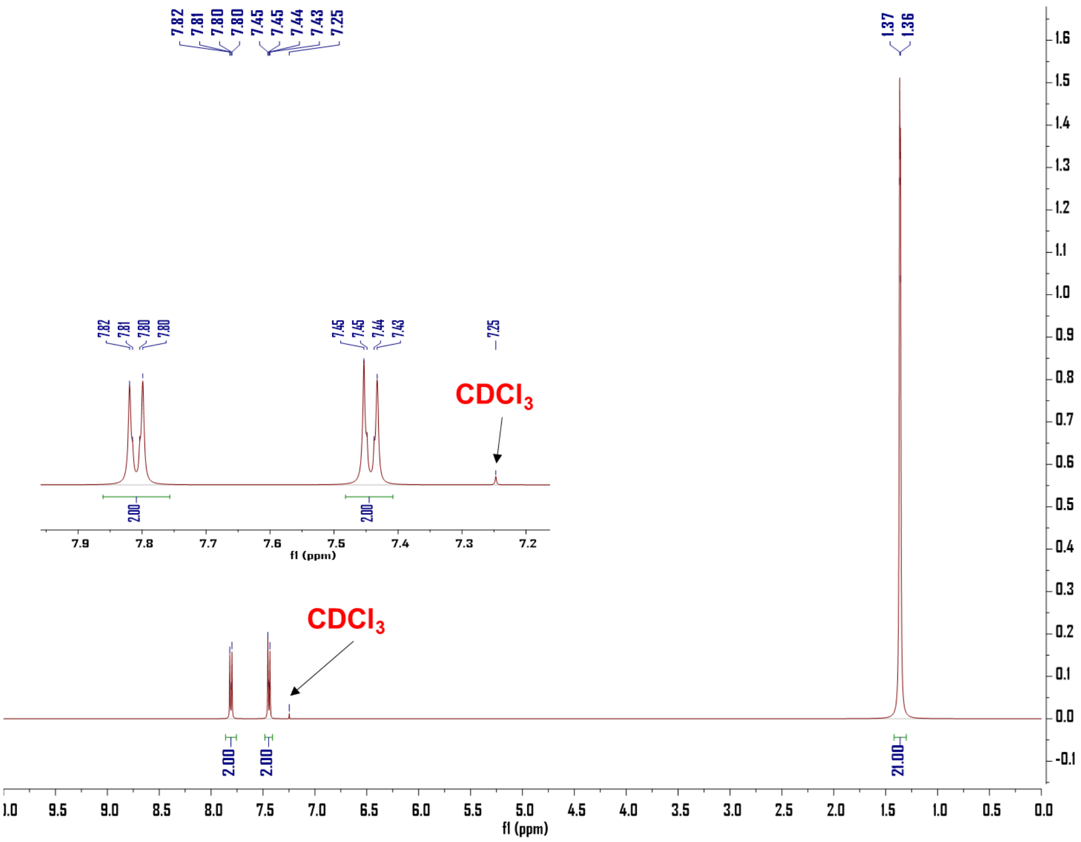

Figure S2. ^{1}H-NMR spectrum of compound (2) in Chloroform-*d*.

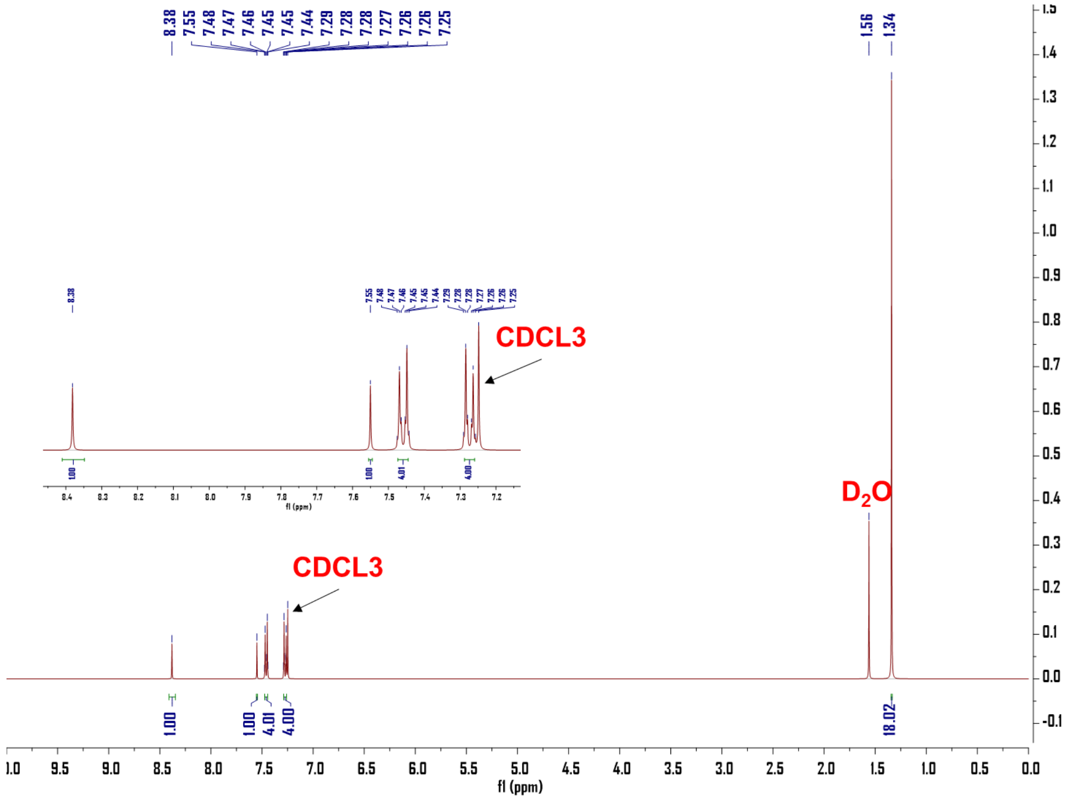

Figure S3. ^{1}H-NMR spectrum of compound (3) in Chloroform-*d*.

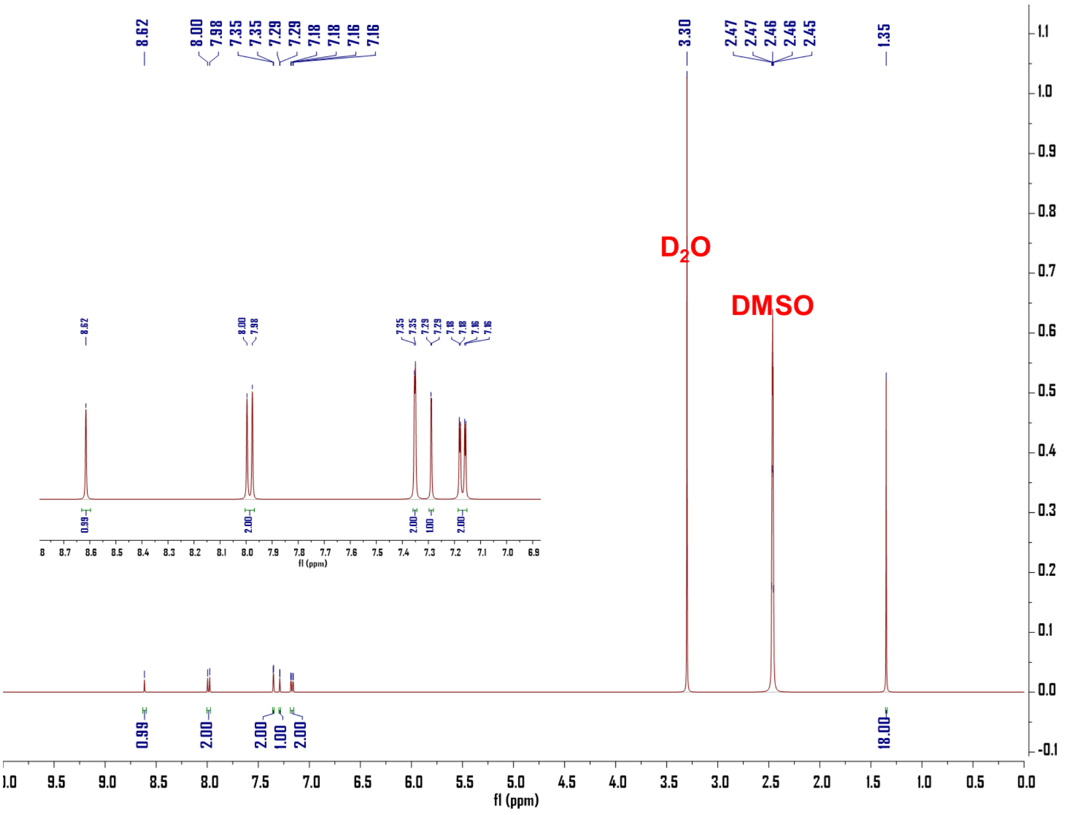

Figure S4. ^{1}H-NMR spectrum of compound (4) in DMSO-d_6.

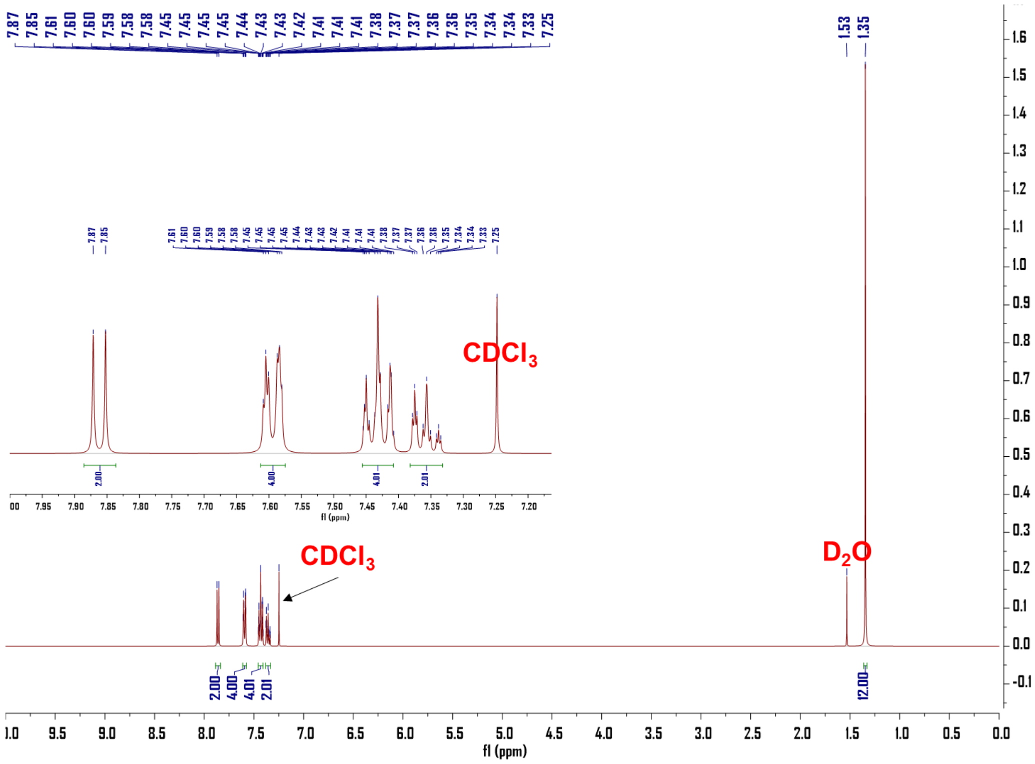

Figure S5. ^{1}H-NMR spectrum of compound (5) in Chloroform-*d*.

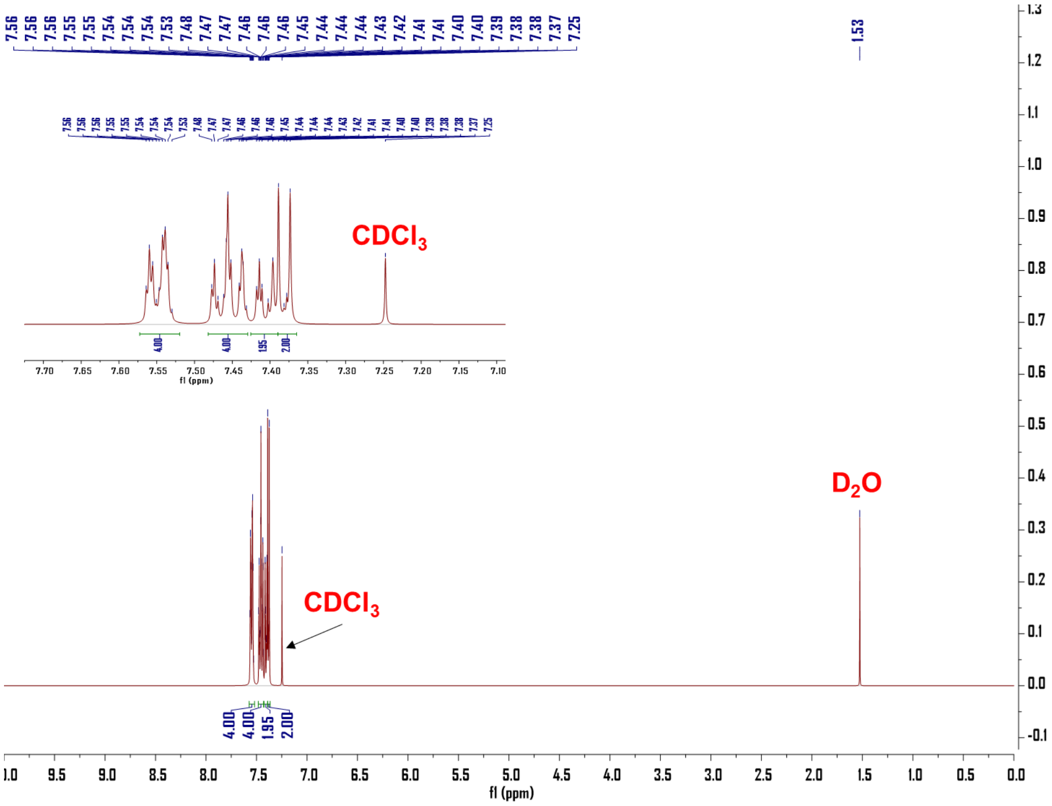

Figure S6. ^{1}H-NMR spectrum of compound (6) in Chloroform-*d*.

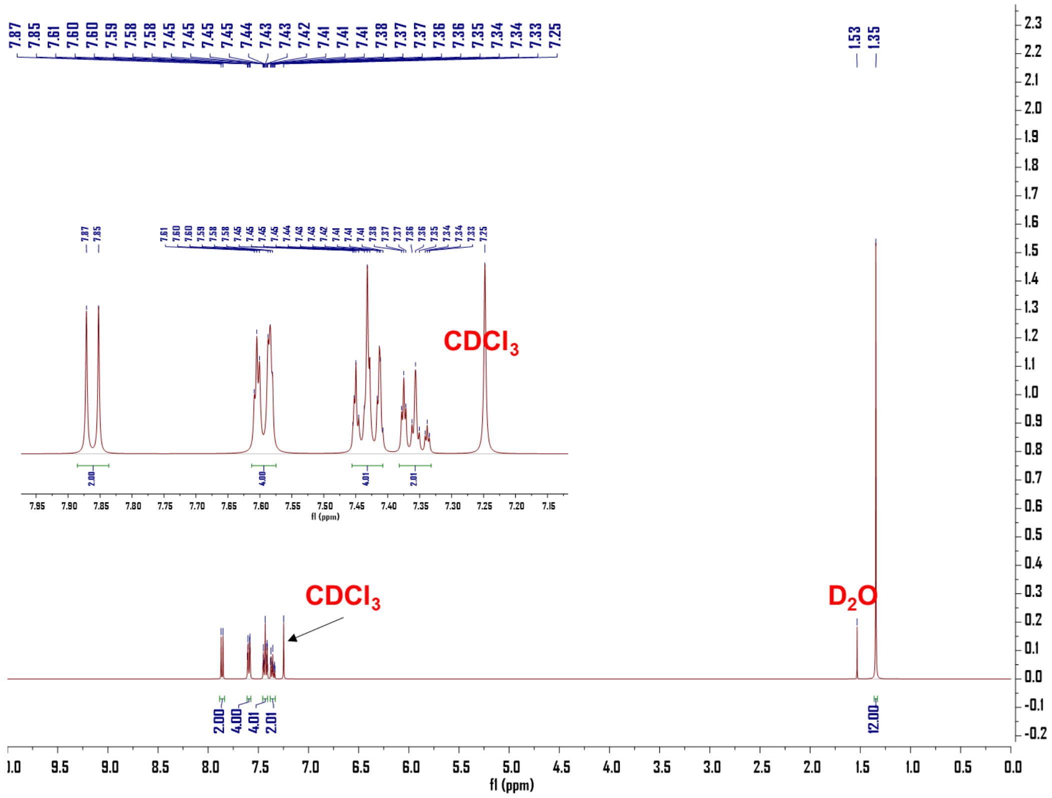

Figure S7. ^{1}H-NMR spectrum of compound (7) in Chloroform-*d*

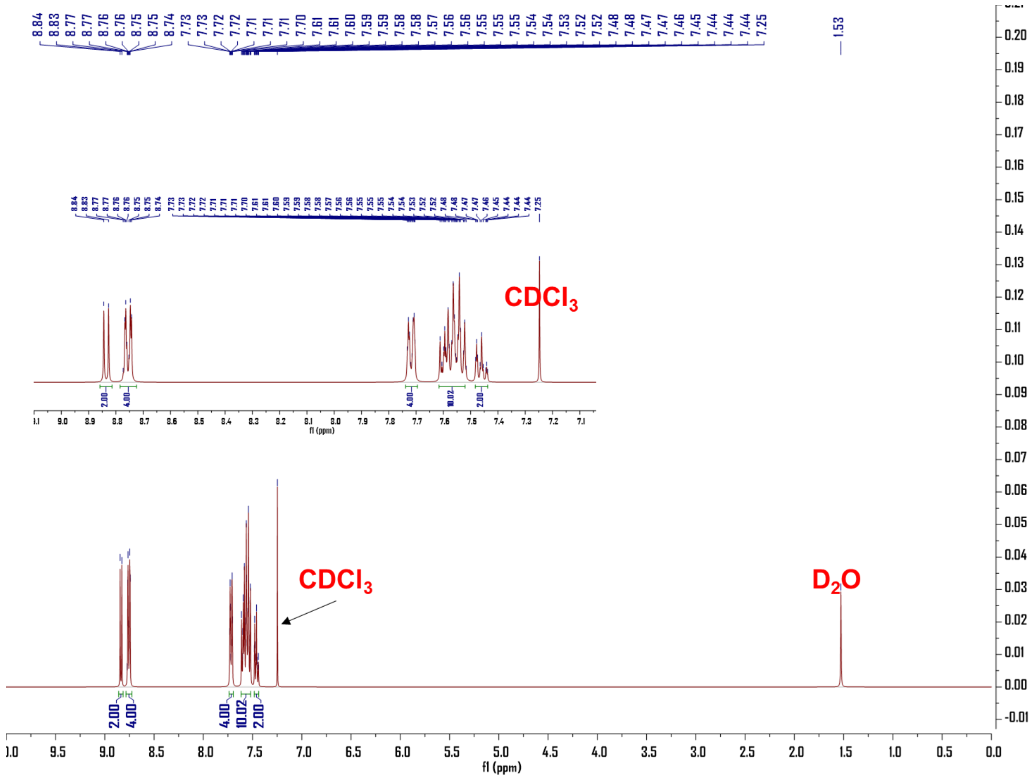

Figure S8. ^{1}H-NMR spectrum of compound (8) in Chloroform-*d*

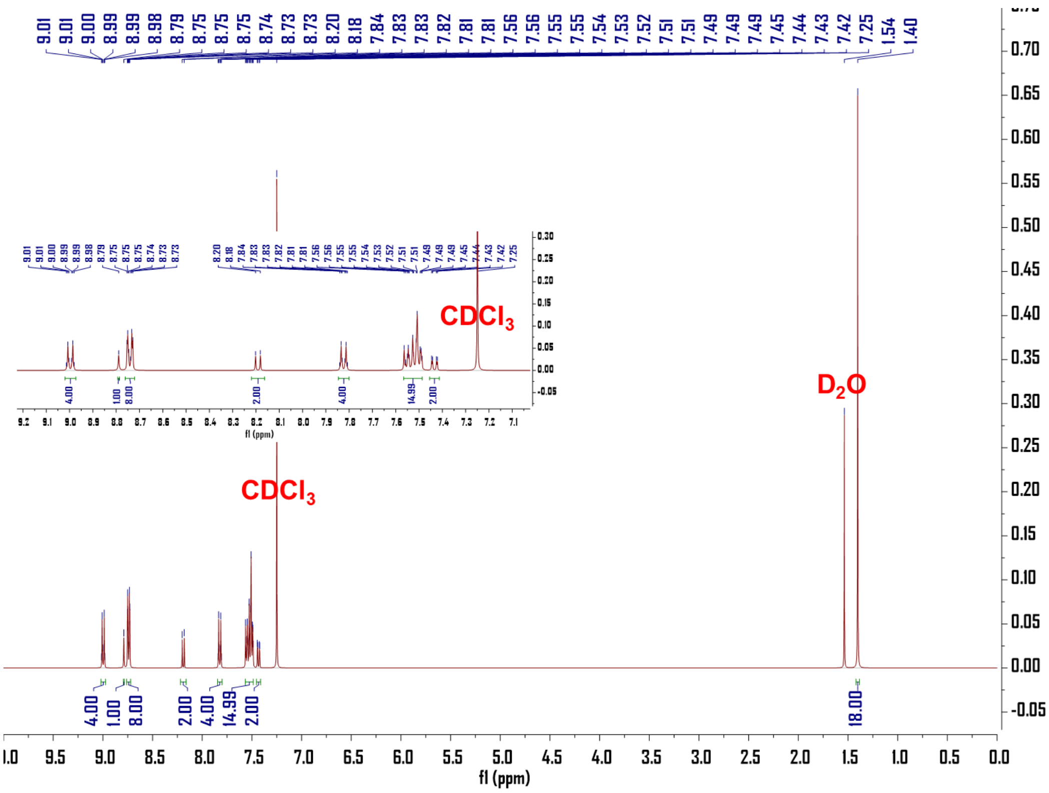

Figure S9. ^{1}H-NMR spectrum of 2TRZ-P-ICz in Chloroform-*d*.

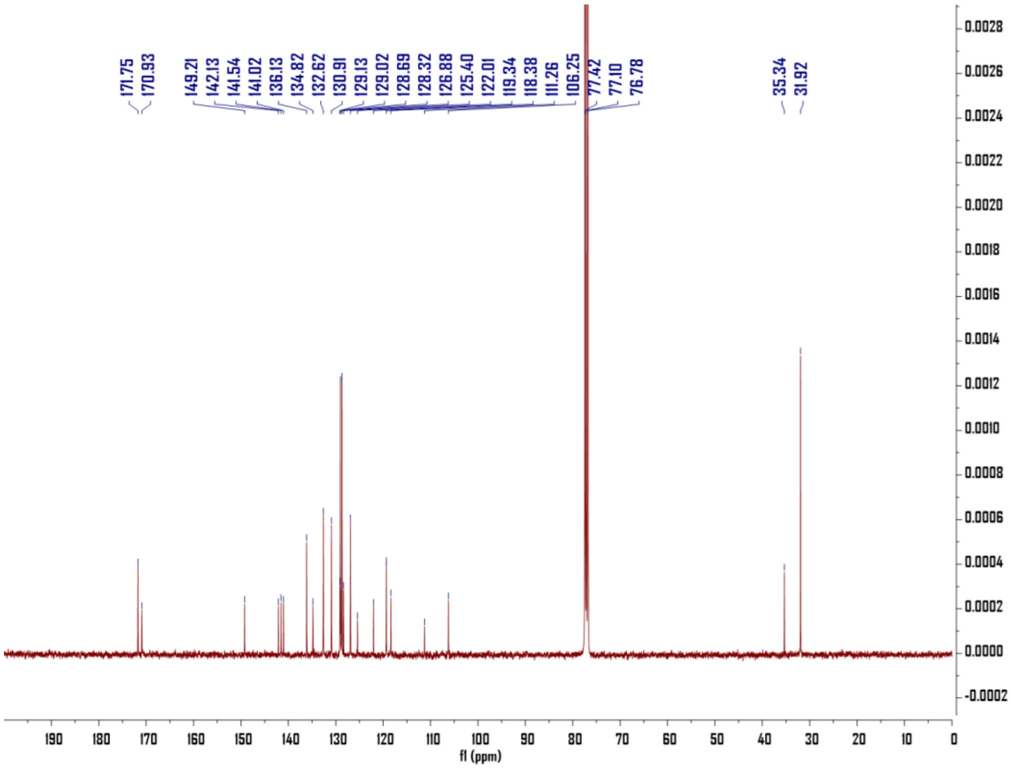

Figure S10. ^{13}C-NMR spectrum of 2TRZ-P-ICz in Chloroform-*d*.

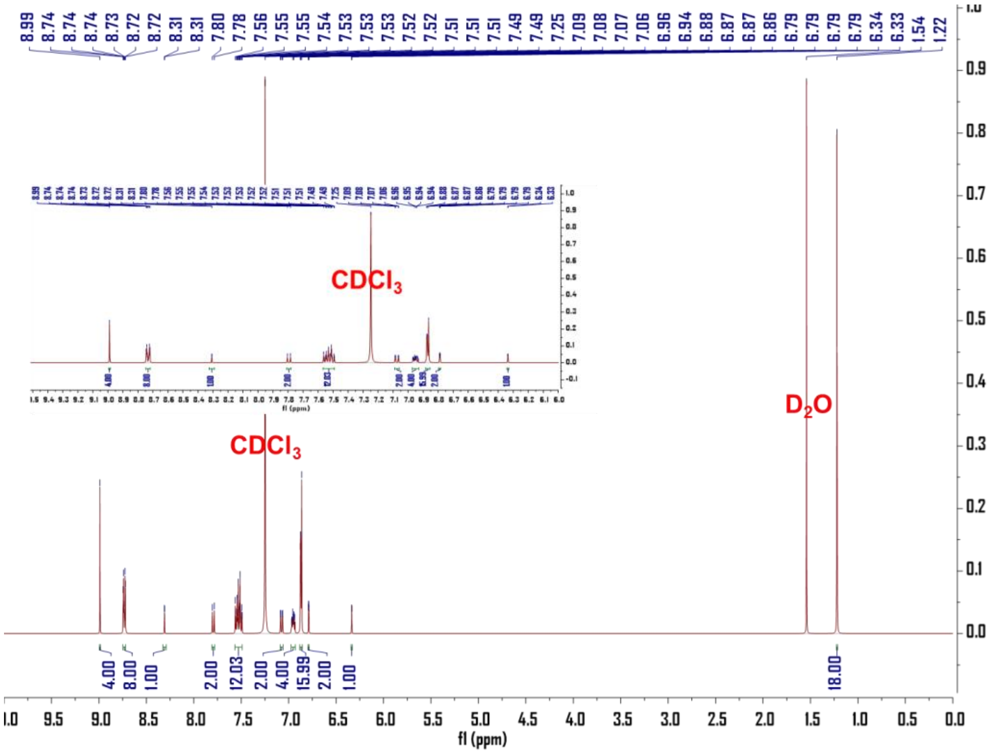

Figure S11. ^{1}H-NMR spectrum of 2TRZ-TP-ICz in Chloroform-*d*.

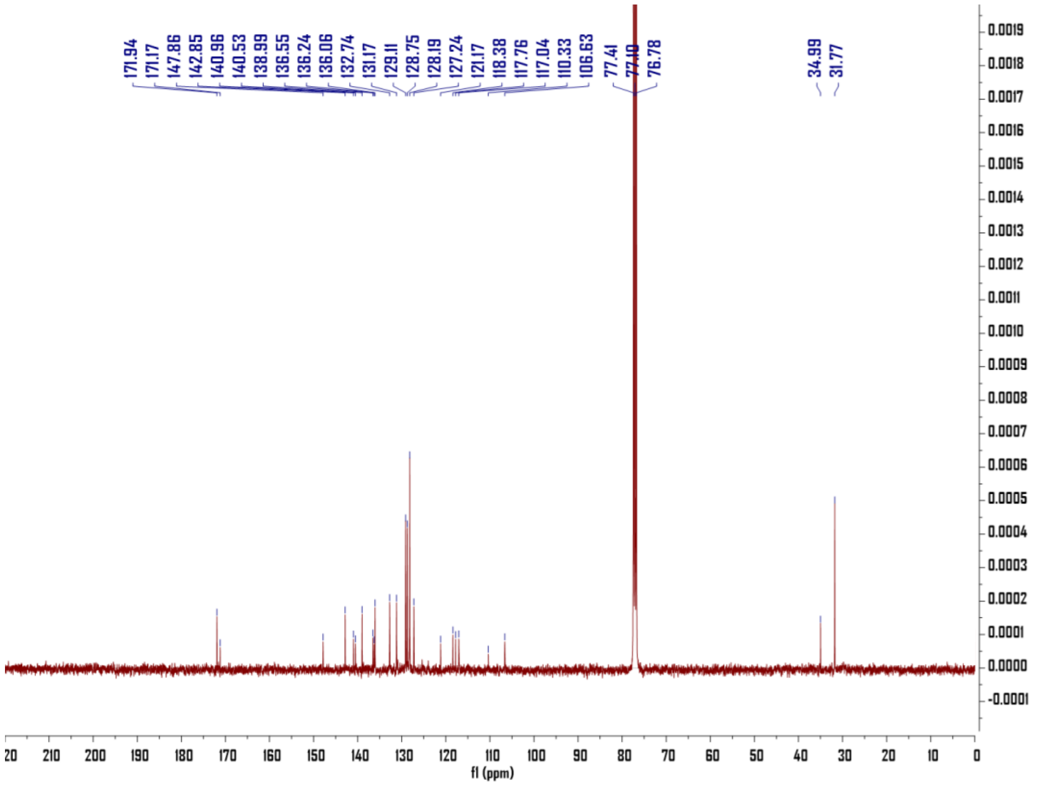

Figure S12. ^{13}C-NMR spectrum of 2TRZ-TP-ICz Chloroform-*d*.

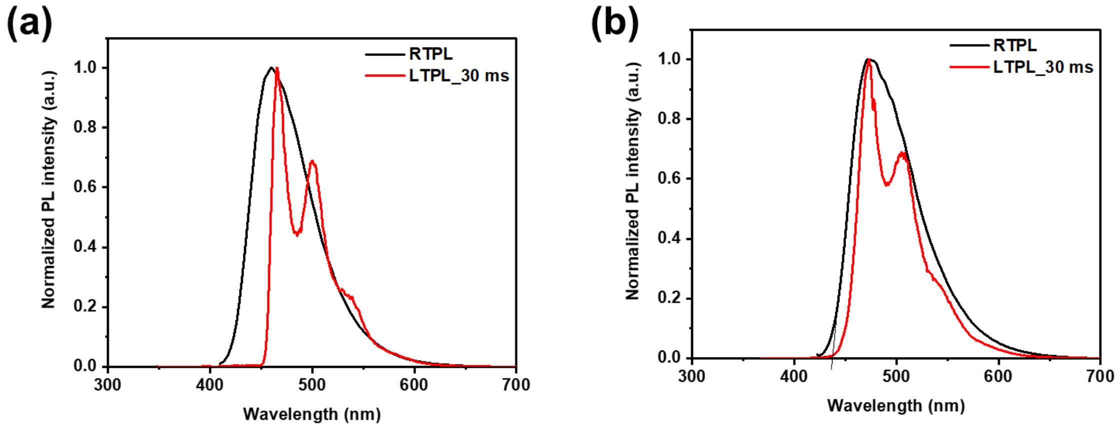

Figure S13. PL spectra acquired under room temperature (RT) and low-temperature (LT) conditions for (a) 2TRZ-P-ICz, and (b) 2TRZ-TP-ICz.

Table S1. Rate constant for the synthesized materials (non-doped film) at room temperature

	2TRZ-P-ICz	2TRZ-TP-ICz
Φ	0.34	0.32
Φ_p	0.33	0.25
Φ_d	0.01	0.07

τ_p(ns)	2.69	22.20
τ_d (µs)	0.188	2.080
k_r (x10^7 1/s)	12.3	1.14
k_{nr} (x10^7 1/s)	20.7	2.52
k_r/k_{nr}	0.593	0.479
k_{IC} (x10^7 1/s)	24.1	2.43
k_{ISC} (x10^9 1/s)	45.3	0.480
Φ_{IC}	0.00527	0.0472
Φ_{ISC}	0.664	0.697
k_{TADF} (x 10^4 1/s)	5.87	4.53
k_{RISC} (x 10^2 1/s)	4.33	32.0

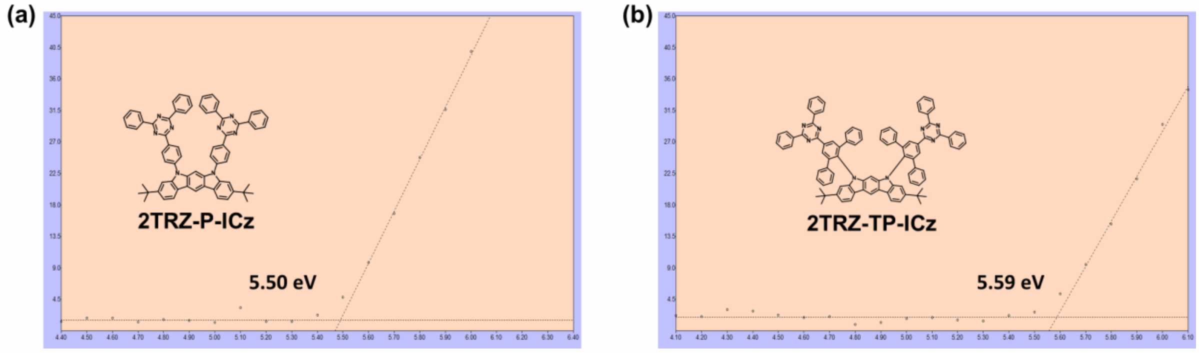

Figure S14. Photoelectron yield spectra (AC2) of (a) 2TRZ-P-ICz and (b) 2TRZ-TP-ICz in neat films.

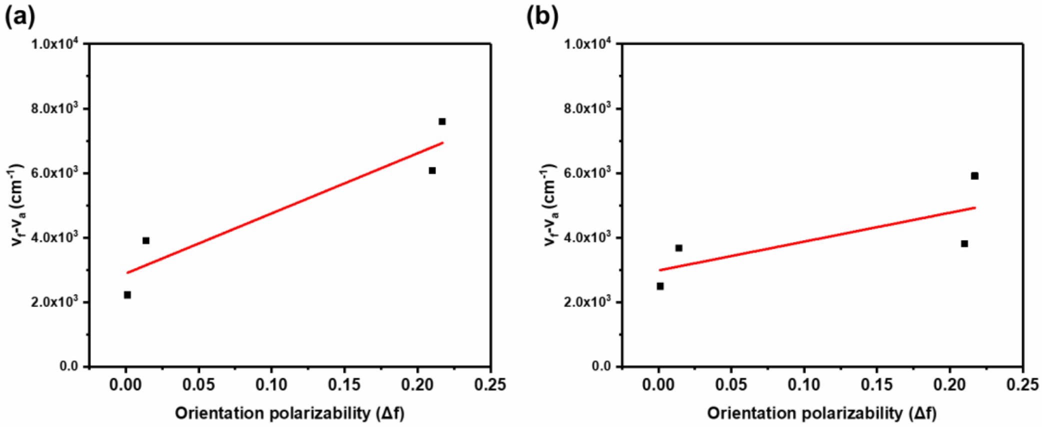

Figure S15. The Lippert–Mataga solvatochromic model of (a) 2TRZ-P-ICz and (b) 2TRZ-TP-ICz.

Table S2. Fitting parameters of Lippert–Mataga plots and Onsager radius (a), $\Delta\mu$ ($\mu_E - \mu_G$) refers to the change in dipole moment.

Compound	Slope (cm^{-1})	a (Å)	$\mu_E - \mu_G$ (D)
2TRZ-P-ICz	18638	7.31	26.9
2TRZ-TP-ICz	8953	7.99	21.3

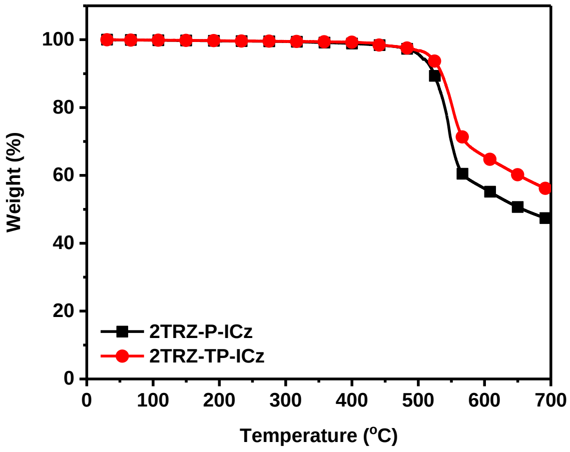

Figure S16. Thermo gravimetric analyzer (TGA) results of the synthesized compounds.

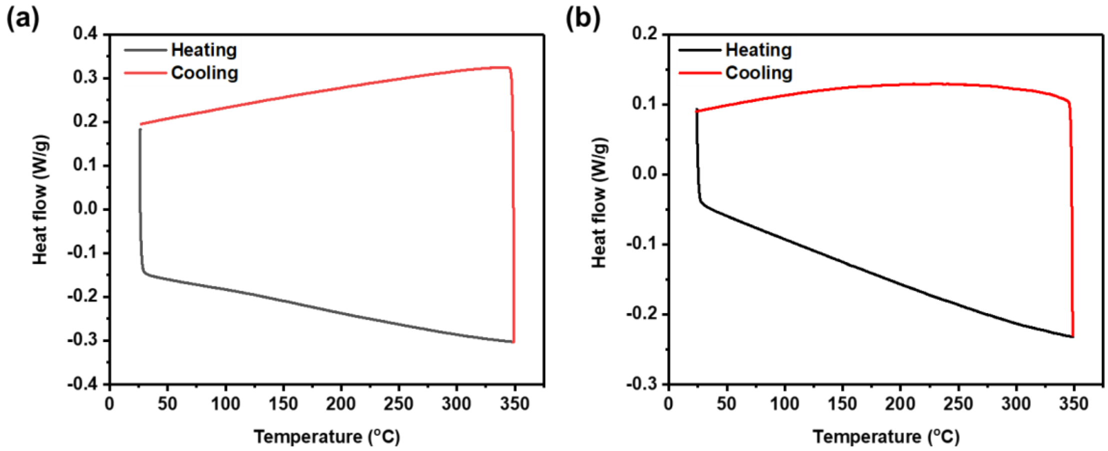

Figure S17. Differential scanning calorimetry (DSC) curves of (a) 2TRZ-P-ICz and (b) 2TRZ-TP-ICz (heating rate: 10 ºC/min, cooling rate: 10 ºC/min).

Table S3. HOMO and LUMO energy levels and band gaps of synthesized materials calculated with B3LYP-D3/def2-TZVPP by ORCA.

B3LYP-D3/ def2-TZVPP	HOMO (eV)	LUMO (eV)	Band gap (eV)
2TRZ-P-ICz	-5.2134	-2.1855	3.0279
2TRZ-TP-ICz	-5.1572	-2.2400	2.9172

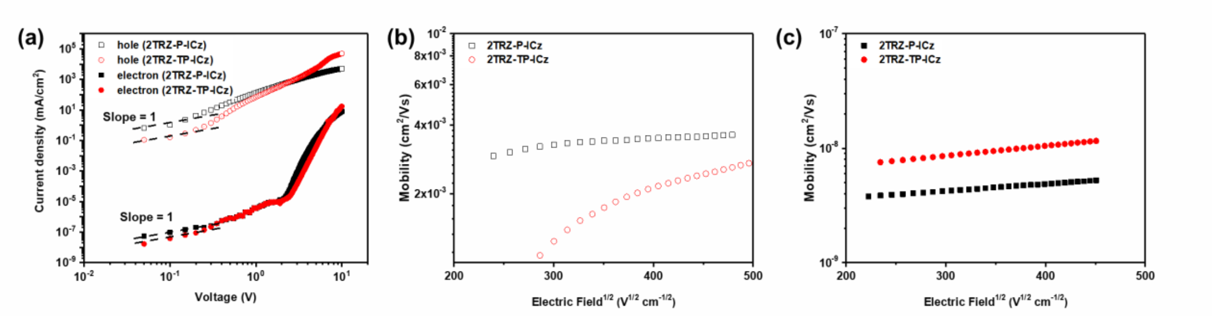

Figure S18. (a) Current density(J)–voltage(V) curves of the hole- and electron-only devices. Plot of (b) hole mobility and (c) electron mobility values as a function of $E^{1/2}$.

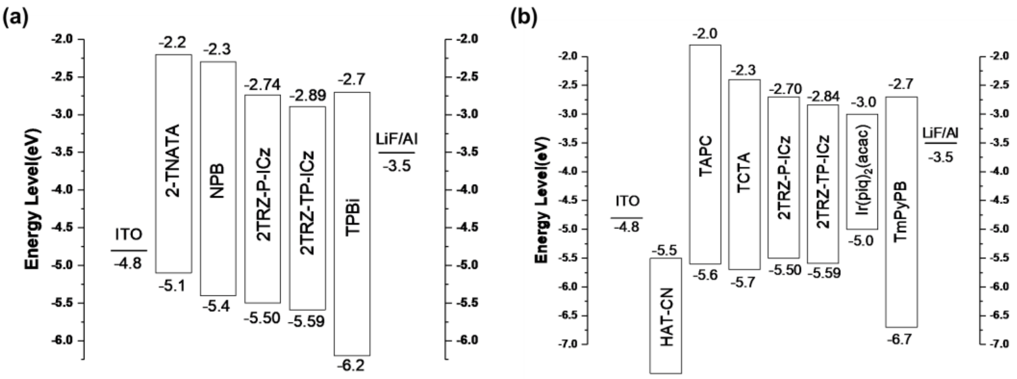

Figure S19. Band diagrams of the fabricated OLED devices: (a) non-doped device, (b) doped device.

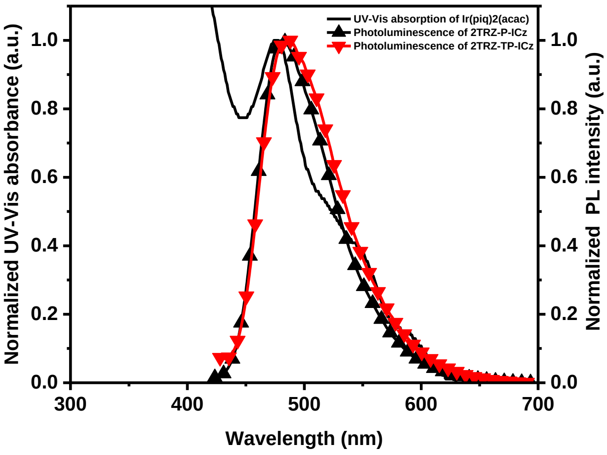

Figure S20. Spectral overlap between $Ir(piq)_2(acac)$ and newly synthesized host materials.

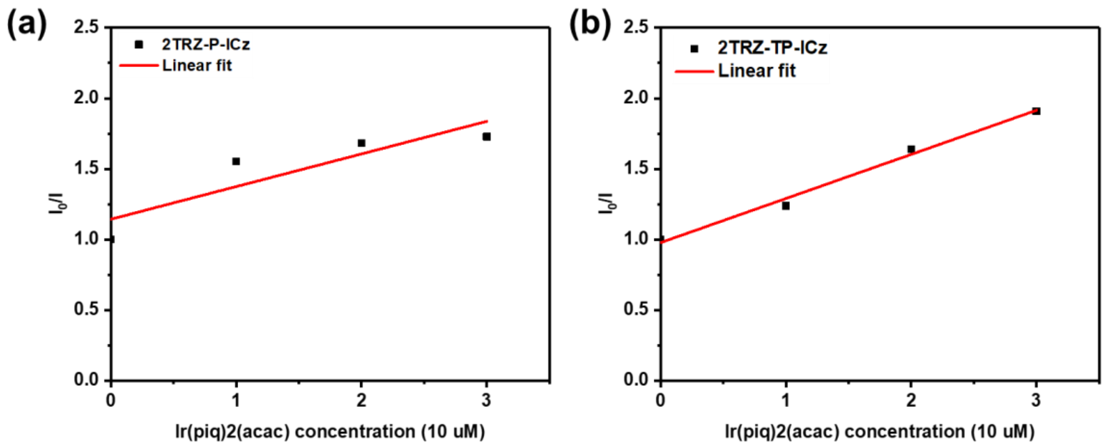

Figure S21. Stern-Volmer plots: (a) 2TRZ-P-ICz, (b) 2TRZ-TP-ICz

Table S4. Rate constant of energy transfer between host and dopant based on Stern-Volmer equation.

	**2TRZ-P-ICz**	**2TRZ-TP-ICz**
Slope	0.23	0.40
k_q[a] [1/s]	1.44×10^7	1.82×10^7

[a]Energy transfer that occurs between host and dopant.

Theoretical value of dipole moment

2TRZ-P-ICz

DIPOLE MOMENT

	X	Y	Z
Electronic contribution:	-0.10863	51.17046	-0.12500
Nuclear contribution :	0.10655	-49.96368	0.12230
	--		
Total Dipole Moment :	-0.00208	1.20678	-0.00270

Magnitude (a.u.) : 1.20679

Magnitude (Debye) : 3.06742

2TRZ-TP-ICz

DIPOLE MOMENT

	X	Y	Z
Electronic contribution :	0.22134	54.59520	-0.29929
Nuclear contribution :	-0.25850	-53.67876	0.29131
	--		
Total Dipole Moment :	-0.03716	0.91644	-0.00798

Magnitude (a.u.) : 0.91723

Magnitude (Debye) : 2.33141
